# Cyclohexyl Ketone Inhibitors of Pin1 Dock in a *Trans*-Diaxial Cyclohexane Conformation

**DOI:** 10.1371/journal.pone.0044226

**Published:** 2012-09-19

**Authors:** Guoyan G. Xu, Carla Slebodnick, Felicia A. Etzkorn

**Affiliations:** Department of Chemistry, Virginia Tech, Blacksburg, Virginia, United States of America; National Cancer Institute at Frederick, United States of America

## Abstract

Cyclohexyl ketone substrate analogue inhibitors (Ac–pSer-Ψ[C = OCH]-Pip–tryptamine) of Pin1, the cell cycle regulatory peptidyl-prolyl isomerase (PPIase), were designed and synthesized as potential electrophilic acceptors for the Pin1 active site Cys113 nucleophile to test a proposed nucleophilic addition-isomerization mechanism. Because they were weak inhibitors, models of all three stereoisomers were docked into the active site of Pin1. Each isomer consistently minimized to a *trans*-diaxial cyclohexane conformation. From this, we hypothesize that Pin1 stretches substrates into a *trans*-pyrrolidine conformation to lower the barrier to isomerization. Our reduced amide inhibitor of Pin1 adopted a similar *trans*-pyrrolidine conformation in the crystal structure. The molecular model of **1**, which mimics the l-Ser-l-Pro stereochemistry, in the Pin1 active site showed a distance of 4.4 Å, and an angle of 31° between Cys113-S and the ketone carbon. The computational models suggest that the mechanism of Pin1 PPIase is not likely to proceed through nucleophilic addition.

## Introduction

Pin1 (peptidyl-prolyl isomerase (PPIase) interacting with never-in-mitosis A kinase-1) was discovered in 1996 as a PPIase enzyme that regulates mitosis [1]. The two domains of Pin1, a WW and a PPIase domain, are connected by a flexible linker that serves as a communication conduit between the domains [2]. Both of these domains recognize the phospho-Ser/Thr-Pro bonds present in mitotic phosphoproteins [3]. Pin1 is distinct from two other PPIase families, cyclophilin and FK506 binding protein (FKBP) [4], since Pin1 only has PPIase activity for phosphorylated substrates [3]. Pin1 catalyzes prolyl cis-trans isomerization to function as a molecular timer regulating the cell cycle, cell signaling, gene expression, immune response, and neuronal function [5]. Pin1 is overexpressed in many cancer lines, and plays an important role in oncogenesis [6]. Because of its significant role in cell cycle regulation by a unique mechanism, Pin1 represents an intriguing diagnostic and therapeutic target for cancer [7], [8]. Several promising classes of Pin1 inhibitors have been synthesized as potential lead compounds [7], including designed inhibitors [9], [10], [11], [12], [13], [14], and natural products [15], [16].

**Figure 1:**
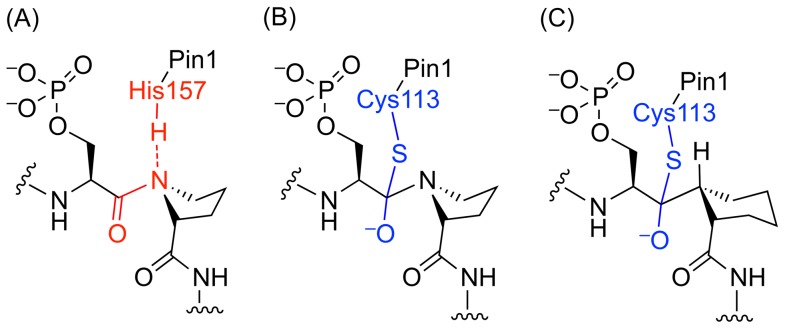
Ketone inhibitors were designed to mimic the tetrahedral intermediate of proposed mechanism B. (A) Proposed Pin1 hydrogen-bond assisted twisted amide mechanism [25], (B) Pin1 Cys113 nucleophilic-addition mechanism tetrahedral intermediate proposed by Ranganathan et al [26]. (C) Electrophilic ketone inhibitor designed to mimic the proposed tetrahedral intermediate upon Cys113-S nucleophilic addition.

The mechanisms of the PPIases, cyclophilins and FKBPs, were shown to go through a twisted amide transition state. Evidence included secondary deuterium isotope effects, molecular modeling, mutagenesis, and bound inhibitor structure [17], [18], [19], [20], [21], [22], [23], [24]. There are two proposed mechanisms for Pin1 catalysis: (1) the twisted-amide mechanism [25], and (2) the nucleophilic-addition mechanism (Figure 1) [26]. In this work, we describe the synthesis, bioassay, and docking of ketones **1**, Ac–l-pSer-Ψ[C = OCH]-l-pipecolyl (Pip)–tryptamine, and ***rac-*** **2**, enantiomeric Ac–d-pSer-Ψ[C = OCH]-l-Pip–tryptamine and Ac–l-pSer-Ψ[C = OCH]-d-Pip–tryptamine. These inhibitors were designed as electrophilic acceptors of the Pin1 active site Cys113 thiol nucleophile to mimic the enzyme-bound tetrahedral intermediate (Figure 1C).

**Figure 2:**
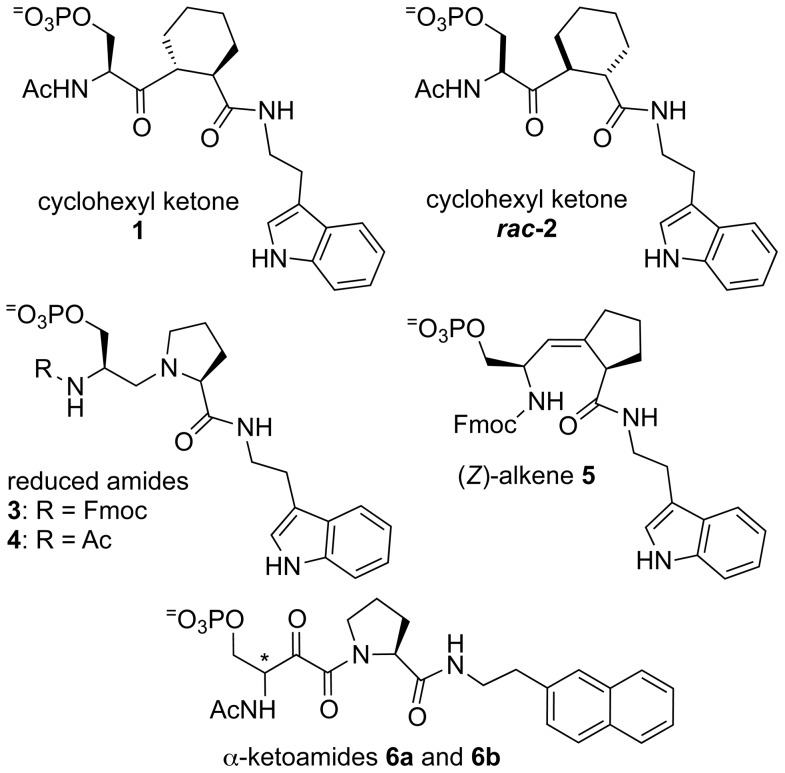
Pin1 inhibitors discussed are cyclohexyl ketones 1 and *rac*-2 (this work); reduced amides 3 and 4 [**27**]; (*Z*)-alkene 5 [**13**]; and α-ketoamides 6a and 6b [**14**].

On the other side of the coin, we have described reduced amides designed as twisted-amide transition-state analogues **3** and **4** (Figure 2) [27]. The evidence for a nucleophilic addition mechanism included the proximity of Cys113 to the substrate in the X-ray crystal structure, and the attenuation of activity for Pin1 mutants: 20-fold for C113S and 120-fold for C113A [26]. We anticipated that the ketones would be poor inhibitors, while the reduced amides, as twisted-amide analogues, would fare better. Indeed, the reduced amide **3** is a better Pin1 inhibitor than a similarly substituted substrate analogue (*Z*)-alkene isostere **5** (Figure 2) [13], [27]. Our crystal structure of reduced amide **4** bound to the Pin1 catalytic site adopted a *trans*-pyrrolidine conformation, supporting the twisted-amide mechanism [27].

Ketones have been widely used as analogues of aldehydes or carboxylic acids to inhibit serine, cysteine [28], [29], and aspartyl proteases [30], [31]. Substrate-analogue ketones have not yet been developed as inhibitors of Pin1. Juglone is a ketone natural product that was shown to be a non-specific inhibitor of Pin1 through Michael addition to a surface Cys thiol of Pin1, resulting in unfolding [15]. Daum et al developed a series of aryl indanyl ketone inhibitors of Pin1; the best inhibitor had an IC_50_ value of 0.2 µM [11]. These inhibitors were reversible and cell penetrating, and they showed biological activities against p53 and β-catenin [11]. Daum et al proposed that the aryl indanyl ketones mimic the transition state of the twisted amide, based on the conformation in a crystal structure [11]. α-Ketoamides **6a** and **6b** were designed as potential transition state analogue inhibitors of Pin1, but their weak inhibition could not be used support either the twisted-amide or the nucleophilic-addition mechanism (Figure 2) [14].

## Results

### Design of Inhibitors

Ketone **1** was designed as a tetrahedral intermediate analogue, incorporating an electrophilic ketone to act as an acceptor for the Pin1 active site Cys113 thiol (Figure 1). Ketone **1** was designed based on substrate and peptide inhibitor specificities [12], [32]. The stereoisomer obtained as a side product during synthesis, ***rac*** **-2**, was also tested for Pin1 inhibition because Wildeman et al. found that d-Thr containing peptide inhibitors were more potent than l-Thr [12]. The carbocyclic analogue of Pip, a cyclohexyl ring, was chosen based on the 100-fold improved inhibition of peptides with a Pip instead of a Pro residue [12], [32]. Tryptamine was coupled to the C-terminus, since Pin1 binds large aromatic residues there [3], [12], [32]. An acetyl was used at the N-terminus because X-ray crystal structures of bound inhibitors showed no electron-density for residues on the N-terminal side of pSer [32], [33]. The acetyl group also improved the water solubility of the inhibitors compared with Fmoc analogues for enzyme assays [13].

### Synthesis

**Figure 3:**
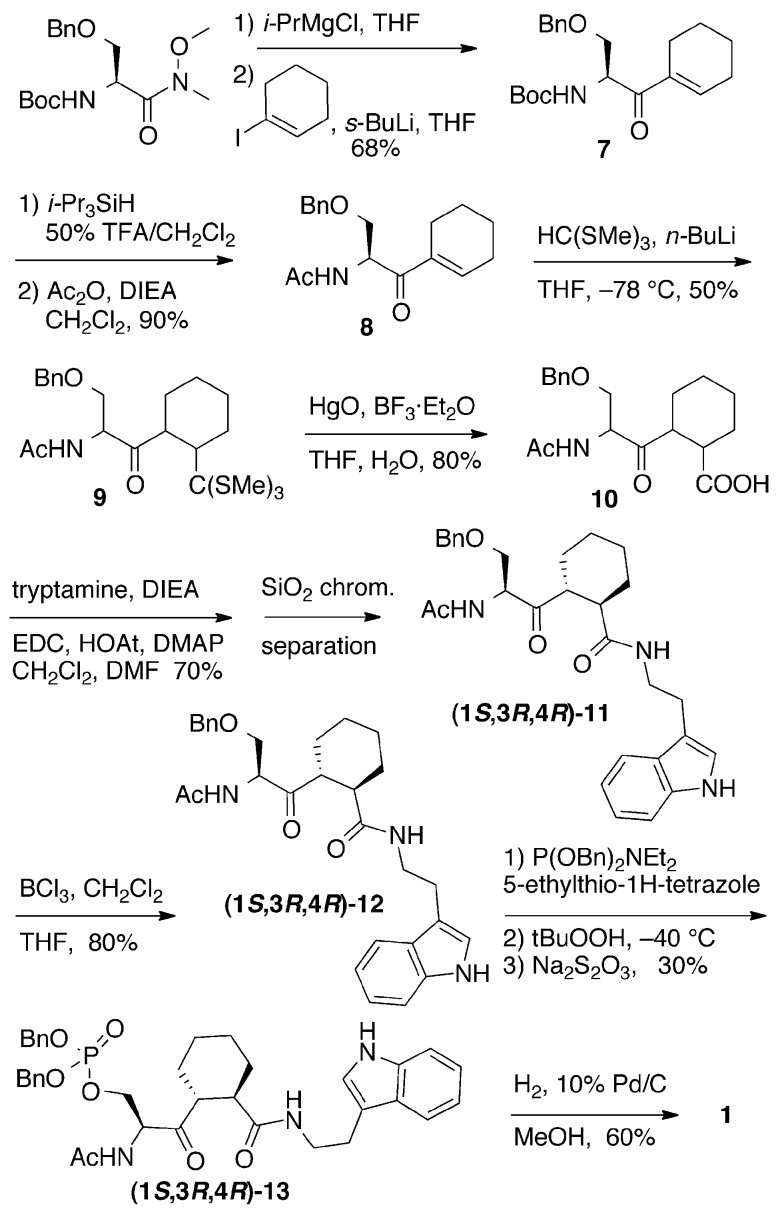
Cyclohexyl ketone inhibitor 1 was synthesized by the method shown.

In the synthesis of ketones **1** and ***rac*** **-2**, addition of cyclohexenyl lithium to a Weinreb amide was used to form the ketone functionality (Figure 3). α,β-Unsaturated ketone **7** was obtained by deprotonation of Boc-Ser(Bn)-N(OMe)Me Weinreb amide with *i*-PrMgCl, followed by addition of cyclohexenyl lithium [34]. The lithium reagent was prepared in situ by treating 1-iodocyclohexene with *s*-BuLi [34], [35].

The Boc group was then removed with TFA, and the amine formed was acetylated with acetic anhydride to give ketone **8** (Figure 3). Michael addition to form orthothioester **9** was accomplished with LiC(SMe)_3_, similar to a synthesis of (+)-methylenolactocin [36]. We first attempted the Michael addition with Boc-protected α, β-unsaturated ketone **7**, however a cyclic carbamate was formed as the major product instead of the desired orthothioester. We have used similar cyclic carbamates in stereochemical proofs [34]. The carbamate ring-closure cannot occur with the acetyl amide. After Michael addition, two major diastereomers of the orthothioester were obtained as a mixture; a minor diastereomer was removed during chromatography. Hydrolysis of orthothioester **9** in a mixture of THF and H_2_O with BF_3_⋅Et_2_O and HgO gave a mixture of diastereomeric carboxylic acids **10** [36]. Without further purification, acids **10** were coupled to tryptamine with EDC to generate the ketone diastereomeric mixture of **(1** ***S*** **,3** ***R*** **,4** ***R*** **)-11** and ***rac*** **-11**, which were separated by silica flash chromatography (Figure 3).

The two diastereomers were carried on separately to the final compounds **1** (Figure 3), and ***rac*** **-2**. The major diastereomer **(1** ***S*** **,3** ***R*** **,4** ***R*** **)-11** was treated with BCl_3_ to remove the benzyl group and form alcohol **(1** ***S*** **,3** ***R*** **,4** ***R*** **)-12** [37], [38]. Phosphorylation with dibenzylphosphoramidite gave dibenzyl phosphate **(1** ***S*** **,3** ***R*** **,4** ***R*** **)-13** [10], [39]. Phosphorylations were also attempted with di-*tert*-butyl or dicyanoethyl phosphoramidites to produce di-*tert*-butyl or dicyanoethyl instead of dibenzyl phosphate. Neither of these phosphates was stable on silica gel, and β-elimination products were obtained after chromatography. TFA deprotection of crude di-*tert*-butyl phosphate, and NH_4_OH deprotection of crude dicyanoethyl phosphate both gave β-elimination products as well. Thus, the dibenzylphosphate was chosen to carry through to the final products **1** and ***rac*** **-2**.

Hydrogenation of the crude dibenzyl phosphate **(1** ***S*** **,3** ***R*** **,4** ***R*** **)-13** went very slowly, giving a complex crude mixture. Thus, **(1** ***S*** **,3** ***R*** **,4** ***R*** **)-13** was purified by reverse-phase semi-preparative high performance liquid chromatography (HPLC). With pure dibenzyl phosphate, hydrogenation at atmospheric pressure worked very well, and gave a very clean final product **1**, similar to our experience with α-ketoamides [14].

### X-ray crystallography

**Figure 4:**
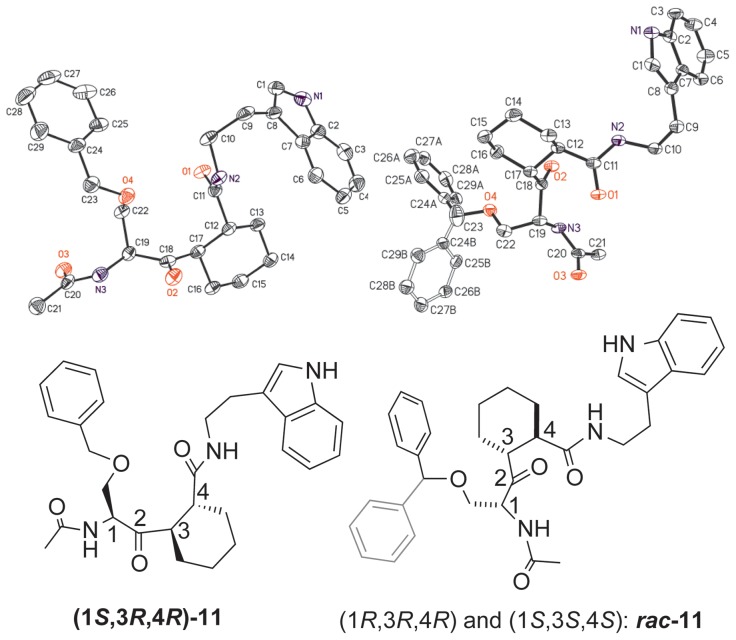
X-ray crystal structures of intermediates (1*S*,3*R*,4*R*)-11 and *rac*-11 are shown above as displacement ellipsoid drawings (50%). The positional disorder of the benzyl group in ***rac*** **-11** is shown as lighter lines. Hydrogen atoms are omitted for clarity. Structural depiction of the stereochemistries of **(1** ***S*** **,3** ***R*** **,4** ***R*** **)-11** and ***rac*** **-11** are shown below each crystal structure.

During the synthesis of the inhibitors, Michael addition of tris-thiomethyl methide to an α,β-unsaturated ketone **8** produced three stereoisomers of **9**, which could not be readily separated (Figure 3). Two diastereomers of a subsequent synthetic intermediate, **(1** ***S*** **,3** ***R*** **,4** ***R*** **)-11** and *rac*-**11**, were separated by chromatography. Each diastereomer was crystallized, and the relative stereochemistry was determined. The absolute configuration of the major diastereomer was assigned to be **(1** ***S*** **,3** ***R*** **,4** ***R*** **)-11**, with the original Ser configuration intact (Figure 4). The minor isomer, ***rac*** **-11**, proved to be a racemic mixture. The absolute configurations were assigned as (1*R,*3*R,*4*R*)-**11** and (1*S,*3*S,*4*S*)-**11**, in which the stereocenter of the Ser analogue was partially epimerized to the *syn*-Ser-*trans*-cyclohexyl configuration (Figure 4).

### Pin1 PPIase Enzyme Assays

The α-chymotrypsin protease-coupled assay was used to evaluate inhibition of Pin1 by compounds **1** and ***rac*** **-2** with the same substrate concentration as described previously [10], [14]. The IC_50_ values of the two diastereomers were determined to be 260±30 µM for **1**, and 61±8 µM for ***rac*** **-2**. Preincubation with Pin1 for 15 minutes did not result in improved inhibition.

### Molecular modeling

**Figure 5:**
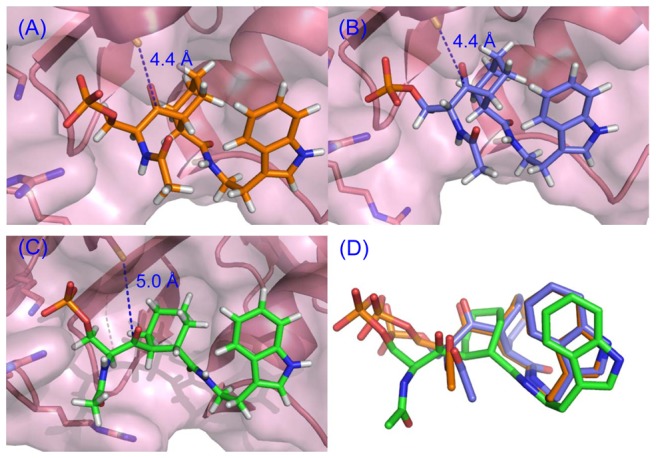
Models of cyclohexyl ketone inhibitors were docked with dynamic minimization. (A) **(1** ***S,*** **3** ***R,*** **4** ***R*** **)-1** in orange, (B) **(1** ***R,*** **3** ***R,*** **4** ***R*** **)-2** in blue, (C) **(1** ***S,*** **3** ***S,*** **4** ***S*** **)-2** in green, and (D) superposition of all atoms of **1** and ***rac*** **-2**. Models were based on PDB 2Q5A [32], and minimized using Sybyl 8.1.1 [42]. Images were prepared using MacPyMol [44].

**Figure 6:**
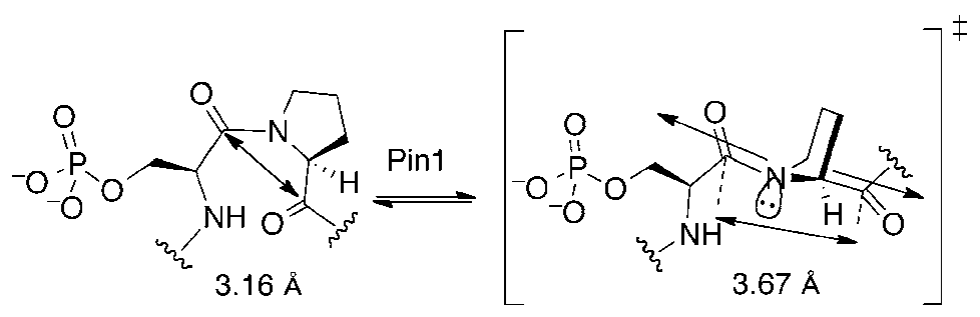
Pin1 is proposed to stretch the prolyl ring by binding phosphate and C-terminal residues tightly, creating a *trans*-pyrrolidine conformation of the substrate and forcing pyramidalization of the prolyl nitrogen in the twisted-amide mechanism. Distance measurements are from calculated structures of Ac–Pro–OH in the ground state and the *trans*-pyrrolidine transition state.

Each of the three cyclohexyl ketone inhibitors was docked flexibly, with geometry minimization, into the Pin1 active site. The resulting docked stereoisomers, (1*S,*3*R,*4*R*)-**1**, (1*R,*3*R,*4*R*)-**2**, and (1*S,*3*S,*4*S*)-**2**, are shown in Figure 5. The total energies, Cys113–S—C = O ketone distances, and angles are reported in Table 1. The distance between 1,2-diequatorial carbonyl groups was 2.93 Å, while the distance between 1,2-diaxial carbonyl groups in 1,2-cyclohexanedial was 3.79 Å after geometry optimization. The distance between the carbonyl carbons of Ac–*cis*-Pro–OH after geometry optimization was 3.16 Å; with the *trans*-pyrrolidine torsion angle fixed during geometry optimization, the distance was 3.67 Å (Figure 6).

**Table 1:** Comparison of cyclohexyl ketone inhibitor-Pin1 complex molecular models.

Stereoisomer:	(1*S,*3*R,*4*R*)-1	(1*R,*3*R,*4*R*)-2	(1*S,*3*S,*4*S*)-2
Mimics:	L-Ser-L-Pro	D-Ser-L-Pro	L-Ser-D-Pro
Color in Figure 5	orange	blue	green
Total E (kcal/mol)	−477	−518	−494
Cys113–S—C = O (Å)	4.4	4.4	5.0
Cys113–S—C = O ∠	31°	102°	59°

## Discussion

### Stereochemical results of inhibitor synthesis

Thermodynamic control in the Michael addition resulted in the *anti*-Ser-*trans*-cyclohexyl stereoisomer of **9** as the major product (Figure 4). The chiral center adjacent to the Ser carbonyl was easily epimerized due to the electron-withdrawing effects of both the α-amide and α-ketone, resulting in an enantiomeric mixture of a second diastereomer, *rac*-**9**. Because the unnatural d-Thr-containing inhibitors were more potent than the l-Thr in work by Zhang et al [32], both diastereomers **1** and ***rac*** **-2** were tested for Pin1 inhibition. Inhibitor **1**, corresponding to the native l-Ser-l-Pro stereochemistry of Pin1 substrates, had an IC_50_ value of 260 µM, while ***rac*** **-2**, an enantiomeric mixture of d-Ser-l-Pro and l-Ser-d-Pro analogues, had an IC_50_ value of 61 µM. Preincubation did not result in improved inhibition, suggesting that they are not slow-binding inhibitors. We obtained a crystal structure of the similarly substituted, reduced amide inhibitor **4** bound in the Pin1 active site, suggesting that the ketones also bind in the active site [27].

### Insights into the Pin1 enzymatic mechanism

To better understand the mechanism of Pin1 PPIase activity, each of the three stereoisomers was docked into the Pin1 active site (Figure 5). Curiously, in each case the inhibitor minimized to a conformation with a trans diaxially substituted cyclohexyl ring. Attempts to force a trans diequatorial conformation on the starting structure resulted in conversion to either a twist boat or a diaxial conformation again. Clearly, the preferred conformation of these cyclohexyl substrate analogues in the Pin1 active site is diaxial. In the crystal structures of intermediates **(1** ***S*** **,3** ***R*** **,4** ***R*** **)-11** and ***rac*** **-11**, the cyclohexyl rings were in the diequatorial chair conformation (Figure 4), which are likely to be the low-energy, solution-phase conformations as well. These inhibitors would thus undergo an unfavorable diequatorial to diaxial conformational change in order to bind to the Pin1 active site.

We hypothesize that the binding interactions of the enzyme with the phosphate and the aromatic group are strong enough to stretch the cyclohexyl rings into the less stable diaxial conformation upon binding (Figure 6). The difference in the distances between diequatorial and diaxial carbonyl groups on a cyclohexane ring was 0.86 Å, an elongation of the structure. The corresponding difference between the planar Ac–*cis*-Pro–OH conformation, and the *trans*-pyrrolidine Ac–Pro–OH conformation was 0.51 Å (Figure 6). This effect of stretching the ring conformation may provide insight into the mechanism of Pin1. In either of the proposed mechanisms: (1) nucleophilic-addition [26], or (2) twisted-amide [25], the nitrogen of the prolyl ring must become pyramidalized and deconjugated from the carbonyl in the transition state [22], [24], [25]. If binding of substrate to the catalytic site forces the Pro ring into a *trans*-pyrrolidine conformation, the nitrogen lone pair and the carbonyl π-bond would no longer be conjugated (Figure 6). The substrate would be destabilized, lowering the barrier to rotation around the amide bond. This proposed stretching action is consistent with the twisted-amide mechanism, providing a more detailed description of how the isomerization might proceed.

Stereoisomer (*R,R,R*)-**2**, with the ketone carbonyl carbon 4.4 Å from the proposed Cys113-S nucleophile, and the S—C = O angle of 102°, had the lowest energy of the three stereoisomers (Table 1). The angle of 102° is close to the optimum angle for nucleophilic addition, i.e. close to the Bürgi-Dunitz angle of 107° [27]. Despite this, the inhibition results suggest that covalent modification, i.e. suicide inhibition, of Pin1 does not occur. Ketones **1** and ***rac*** **-2** were designed as tetrahedral-intermediate analogues based on the nucleophilic-addition mechanism; they do not appear to behave as such. The IC_50_ values are in the range of substrate analogue inhibitors. These results argue against the proposed nucleophilic-addition mechanism for Pin1 [14].

### Stereochemical effects on inhibition

The stereochemistry affected the inhibition, since the racemate ***rac*** **-2** was about 4-fold more potent than diastereomer **1**. Molecular modeling provides insight into the stereochemical preferences of the Pin1 active site. The relative (not absolute) energies of the three models can be compared because they are all stereoisomers bound into the same Pin1 active site (Table 1). These inhibitors are substituted with tryptamine, comparable to our ground-state alkene isostere inhibitor **5** with an IC_50_ value of 25 µM (Figure 2) [13], and with Ac and naphthylethylamine comparable to our α-ketoamide inhibitors **6**, with IC_50_ values of 100 and 200 µM [14]. The Pin1-**(** ***S,R,R*** **)-1** complex, with an intermediate energy, corresponds to the native l-Ser-l-Pro configuration, yet it had very poor inhibition (260 µM), comparable to the similarly substituted α-ketoamides **6** [14]. The Pin1-**(** ***S,S,S*** **)-2** complex, which corresponds to the l-Ser-d-Pro configuration, had the highest energy of the three, while Pin1-**(** ***R,R,R*** **)-2**, corresponding to a d-Ser-l-Pro configuration had the lowest energy. This is consistent with the d-Thr-l-Pip in the most potent peptide inhibitors of Pin1 [12], [32]. We expect that **(** ***R,R,R*** **)-2** isomer would be more potent than the IC_50_ value of 61 µM for ***rac*** **-2** indicates, and **(** ***S,S,S*** **)-2** is likely to be less potent than 61 µM, because the IC_50_ value represents a weighted average of the two. The most potent that either enantiomer could possibly be is 30 µM if the other was not an inhibitor at all. This is highly unlikely, but it serves to show that these ketone inhibitors behave as substrate analogues.

## Conclusions

Three stereoisomeric ketone analogues of Pin1 substrates were synthesized, modeled, and assayed as Pin1 inhibitors. Molecular modeling shows that the inhibitors have a preference for *trans*-diaxial-cyclohexane conformations upon binding to Pin1. This led us to propose a stretching mechanism to attain pyramidalization of the prolyl nitrogen, consistent with the preferred twisted-amide mechanism [25]. The molecular models of the three stereoisomers in the active site of Pin1 confirmed the stereochemical preferences of Pin1 for inhibitors seen in other inhibitors [12], [14], [27], [32]. We attribute the weaker binding of these inhibitors to a combination of: (1) the conformational change required for binding, and (2) the inability of these ketones to act as electrophilic acceptors for the Pin1 Cys113 thiol. The weak inhibition of the ketones, and the correspondingly stronger inhibition by similarly substituted reduced amide inhibitors [27], provides evidence against the nucleophilic addition mechanism for Pin1.

## Materials and Methods

### Synthesis

Unless otherwise indicated, all reactions were carried out under dry N_2_ in flame-dried glassware. THF was distilled from Na-benzophenone, and CH_2_Cl_2_ was dried by passage through dry alumina. Anhydrous DMF (99.8%), MeOH, and DIEA were used directly from sealed bottles. Brine (NaCl), Na_2_S_2_O_3_, NaHCO_3_, and NH_4_Cl refer to saturated aqueous solutions, and HCl refers to a 1 N aqueous solution, unless otherwise noted. Flash chromatography was performed on 230–400 mesh silica gel with reagent grade solvents. Analytical HPLC were obtained on a 4.6×50 mm C18 column with 10% CH_3_CN/H_2_O for 3 min followed by a 10% to 90% CH_3_CN/H_2_O gradient over 6 min unless otherwise noted. HPLC results are reported as retention time, integrated % purity. ^1^H, ^13^C, and ^31^P NMR spectra were obtained at ambient temperature in CDCl_3_, unless otherwise noted. Chemical shifts are reported in parts per million (ppm) downfield from tetramethylsilane (TMS). Data are reported as follows: chemical shift, multiplicity: singlet (s), doublet (d), triplet (t), multiplet (m), broad singlet (br s), coupling constants *J* in Hz, and integration. HPLC chromatograms for compounds **1** and ***rac*** **-2**, ^1^H (500 MHz), ^13^C (125 MHz), and ^31^P NMR (162 MHz) NMR spectra of compounds **1**, ***rac*** **-2**, and **7**–**13**, are available in Dataset S1.

#### Boc-ketone 7

To a solution of 1-iodocyclohexene [35] (5.50 g, 26.4 mmol) in THF (60 mL) at −40°C was added *sec*-butyl lithium (1.4 M in cyclohexane, 37.8 mL, 52.9 mmol). The mixture was stirred at −40°C for 3 h. Boc–Ser(OBn)–N(OMe)Me [34] Weinreb amide (5.96 g, 17.6 mmol) was dissolved in THF (60 mL) in another round-bottom flask and cooled to −78°C, *i*-PrMgCl (2.0 M in THF, 8.64 mL, 17.3 mmol) was then added dropwise. The Weinreb amide solution was stirred at −78°C for 1 h. The cyclohexenyl lithium was added via canula at −78°C to the Weinreb amide solution. After stirring at −78°C for 1 h, the reaction was warmed to rt, stirred for 12 h, and quenched with NH_4_Cl (80 mL). The resulting mixture was diluted with water (40 mL) and EtOAc (100 mL). The aqueous layer was extracted with EtOAc (100 mL). The organic layers were combined, and washed with NH_4_Cl (2×80 mL), NaHCO_3_ (80 mL), and brine (80 mL). The organic layer was dried over Na_2_SO_4_, filtered and evaporated. The crude product was purified by chromatography on silica (eluant: 8% EtOAc/hexanes) to yield ketone **7** (4.3 g, 68%) as a colorless oil. Anal. HPLC, 254 nm, 7.3 min, 98.2%; ^1^H NMR δ 7.28 (m, 5H), 6.91 (m, 1H), 5.59 (d, *J* = 8.3, 1H), 5.13 (dt, *J* = 4.4, 8.3, 1H), 4.54 (d, *J* = 12.4, 1H), 4.42 (d, *J* = 12.4, 1H), 3.68 (dd, *J* = 4.4, 9.3, 1H), 3.66 (dd, *J* = 4.4, 9.6, 1H), 2.36 (m, 1H), 2.21 (m, 2H), 2.12 (m, 1H), 1.62 (m, 4H), 1.44 (s, 9H); ^13^C NMR δ 197.8, 155.5, 141.8, 137.8, 137.4, 128.4, 127.7, 127.6, 79.8, 73.1, 71.3, 54.3, 28.4, 26.2, 23.4, 21.8, 21.5; ESI^+^ HRMS m/z 382.1998 [M+Na]^+^. Calculated for C_21_H_29_NO_4_·Na 382.1994.

#### Acetyl-ketone 8

Boc-ketone **7** (1.5 g, 4.2 mmol) was dissolved in CH_2_Cl_2_ (20 mL), and *i*Pr_3_SiH (0.2 mL) and TFA (20 mL) were added. The mixture was stirred at rt for 0.5 h. The reaction mixture was then concentrated under reduced pressure. The residue was triturated with hexanes (3×25 mL). After evaporation in vacuo for 2 h, the ammonium salt obtained was dissolved in CH_2_Cl_2_ (20 mL), and Ac_2_O (2 mL) and DIEA (2 mL) were added. The reaction mixture was stirred at rt for 1 h. After dilution with CH_2_Cl_2_ (30 mL), the mixture was washed with HCl (2×25 mL), 1 N NaOH (2×25 mL), and brine (25 mL). The organic layer was dried over Na_2_SO_4_, filtered and evaporated. The residue was purified by flash chromatography on silica (step gradient: 25% then 50% EtOAc/hexanes) to yield **8** (1.1 g, 90%) as a pale, yellow oil. Anal. HPLC, 254 nm, 5.1 min, 100%; ^1^H NMR δ 7.35-7.20 (m, 5H), 6.93 (m, 1H), 6.62 (br, 1H), 5.42 (m, 1H), 4.52 (d, *J* = 12.3, 1H), 4.40 (d, *J* = 12.3, 1H), 3.70 (m, 2H), 2.39-2.04 (m, 4H), 2.02 (s, 3H), 1.62 (m, 4H); ^13^C NMR δ 197.3, 169.8, 142.3, 137.7, 137.2, 128.5, 127.9, 127.6, 73.2, 71.1, 53.3, 26.2, 23.44, 23.38, 21.8, 21.5; ESI^+^ HRMS m/z 302.1760 [M+H]^+^. Calculated for C_18_H_24_NO_3_. 302.1756.

#### Orthothioformate 9

*n*-Butyl lithium (2.5 M in hexane, 6.81 mL, 17.0 mmol) was added dropwise to a solution of CH(SMe)_3_ (2.68 g, 17.0 mmol) dried over 4 Å molecular sieves in THF (65 mL) at −78°C. The solution was stirred at −78°C for 2 h. A solution of the acetyl ketone **8** (0.790 g, 2.62 mmol) dried over 4 Å molecular sieves in THF (50 mL) was added dropwise via canula. The reaction mixture was stirred at −78°C for 2 h, and quenched with NH_4_Cl (80 mL). The resulting mixture was extracted with EtOAc (3×150 mL). The organic layer was dried over Na_2_SO_4_, filtered and evaporated. The crude product was purified by chromatography on silica (step gradient: 0% then 20% EtOAc/hexanes) to yield the orthothioformate **9**, a mixture of two diastereomers, (0.60 g, 50%) as a colorless oil. The mixture was used in the next reaction without separation. The major diastereomer was partially separated for characterization. Major diastereomer: ^1^H NMR δ 7.30 (m, 5H), 6.60 (d, *J* = 7.5, 1H), 5.26 (ddd, *J* = 3.4, 4.2, 7.6, 1H), 4.52 (d, *J* = 12.1, 1H), 4.49 (d, *J* = 11.8, 1H), 3.89 (dd, *J* = 3.3, 10.2, 1H), 3.79 (dd, *J* = 4.4, 9.9, 1H), 3.11 (ddd, *J* = 3.8, 10.5, 11.5, 1H), 2.39 (ddd, *J* = 3.6, 10.4, 11.8), 2.09 (m, 1H), 2.07 (s, 9H), 2.02 (s, 3H), 1.82 (m, 1H), 1.73 (m, 1H), 1.63 (m, 2H), 1.38 (m, 1H), 1.31 (m, 1H), 1.22 (m, 1H), 1.06 (m, 1H); ^13^C NMR δ 207.0, 169.4, 137.9, 128.5, 127.9, 127.8, 75.8, 73.4, 68.9, 59.5, 51.5, 48.0, 31.6, 28.6, 25.6, 25.1, 23.6, 15.2; ESI^+^ HRMS m/z 478.1530 [M+Na]^+^. Calculated for C_22_H_33_NO_3_S_3_·Na 478.1520.

#### Ac–Ser(OBn)–Ψ[C = OCH]–2-(indol-3-yl)-ethylamine (1*S*,3*R*,4*R*)-11 and *rac*-11

A mixture of compound **9** (0.30 g, 0.66 mmol) and HgO (0.70 g, 3.2 mmol) was suspended in 4∶1 THF∶H_2_O (45 mL), and BF_3_·Et_2_O (1.2 mL, 9.6 mmol) was added. The mixture was stirred at rt for 3 h. The reaction mixture was diluted with water (10 mL) and extracted with EtOAc (3×50 mL). The organic layer was dried over Na_2_SO_4_, filtered and evaporated. The residue was filtered through silica to remove HgO, and the solvent was evaporated in vacuo. The crude carboxylic acid **10** was dissolved in a mixture of CH_2_Cl_2_ (100 mL) and DMF (15 mL), and tryptamine (0.27 g, 1.7 mmol), EDC (0.32 g, 1.68 mmol), HOAt (0.25 g, 1.6 mmol), DMAP (50 mg, 0.4 mmol) and DIEA (0.37 g, 2.9 mmol) were added. The reaction was stirred at rt for 16 h. The mixture was diluted with EtOAc (400 mL), washed with water (3×150 mL), HCl (3×150 mL), NaHCO_3_ (3×150 mL) and brine (150 mL). The crude product was purified by chromatography on silica (step gradient: 0% then 30% EtOAc/hexanes) to yield two diastereomers. The major diastereomer (120 mg, 36%) and the minor diastereomer (60 mg, 18%) were obtained as colorless oils at first, which then solidified. Both solids were recrystallized from EtOAc∶hexanes (1∶2) to determine the relative stereochemistry by X-ray crystallography.

Major isomer **(1** ***S*** **,3** ***R*** **,4** ***R*** **)-11**: Anal. HPLC, 254 nm, 5.0 min, 98.9%; ^1^H NMR δ 8.10 (br, 1H), 7.57 (dd, *J* = 0.7, 7.8, 1H), 7.37 (dt, *J* = 0.8, 8.2, 1H), 7.31-7.21 (m, 5H), 7.20 (dt, *J* = 1.2, 7.5, 1H), 7.11 (dt, *J* = 0.9, 7.5, 1H), 7.02 (d, *J* = 2.2, 1H), 6.30 (d, *J* = 7.5, 1H), 5.60 (t, *J* = 5.8, 1H), 4.84 (dt, *J* = 4.2, 7.4, 1H), 4.58 (d, *J* = 11.8, 1H), 4.48 (d, *J* = 11.8, 1H), 3.86 (dd, *J* = 3.9, 9.9,1H), 3.76 (dd, *J* = 4.4, 9.9, 1H), 3.44 (m, 2H), 3.05 (ddd, *J* = 3.3, 10.7, 12.4, 1H), 2.84 (m, 2H), 2.37 (m, 1H), 2.13 (m, 1H), 1.99 (s, 3H), 1.80 (m, 3H), 1.49 (m, 1H), 1.26 (m, 2H), 1.08 (m, 1H); ^13^C NMR δ 209.5, 174.7, 170.0, 137.9, 136.5, 128.5, 128.0, 127.9, 127.4, 122.4, 122.3, 119.6, 118.9, 113.1, 111.3, 73.3, 68.4, 60.0, 49.2, 45.8, 39.4, 29.7, 29.5, 25.7, 25.4, 25.3, 23.3; ESI^+^ HRMS m/z 512.2530 [M+Na]^+^. Calculated for C_29_H_35_N_3_O_4_·Na 512.2525. Minor isomer ***rac*** **-11**: Anal. HPLC, 254 nm, 5.1 min, 96.3%; ^1^H NMR δ 8.25 (br, 1H), 7.56 (d, *J* = 8.0, 1H), 7.36 (d, *J* = 8.3, 1H), 7.35-7.25 (m, 5H), 7.19 (ddd, *J* = 1.1, 7.2, 8.1, 1H), 7.11 (ddd, *J* = 1.0, 7.4, 8.0, 1H), 7.00 (d, *J* = 2.2, 1H), 6.75 (d, *J* = 7.7, 1H), 5.56 (t, *J* = 5.8, 1H), 4.86 (dt, *J* = 4.0, 7.4, 1H), 4.49 (d, *J* = 12.0, 1H), 4.46 (d, *J* = 12.1, 1H), 3.92 (dd, *J* = 3.3, 9.9, 1H), 3.75 (dd, *J* = 4.1, 9.9, 1H), 3.48 (q, *J* = 6.4, 2H), 3.01 (dt, *J* = 3.0, 11.1, 1H), 2.86 (m, 2H), 2.33 (m, 1H), 2.01 (s, 3H), 1.78 (m, 4H), 1.43 (m, 1H), 1.19 (m, 3H); ^13^C NMR δ 210.9, 174.5, 170.1, 137.8, 136.5, 128.5, 127.9, 127.88, 127.4, 122.4, 122.2, 119.5, 118.8, 112.9, 111.4, 77.4, 73.4, 68.5, 58.8, 47.8, 47.7, 39.6, 30.0, 28.6, 25.3, 25.1, 23.4; ESI^+^ HRMS m/z 490.2710 [M+H]^+^. Calculated for C_29_H_36_N_3_O_4_ 490.2706.

#### Ac–Ser–Ψ[C = OCH]–Pip–2-(indol-3-yl)-ethylamine (1*S*,3*R*,4*R*)-12 and *rac-12*

Ac–Ser(OBn)–Ψ[C = OCH]–2-(indol-3-yl)-ethylamine **(1** ***S*** **,3** ***R*** **,4** ***R*** **)-11** (48 mg, 0.098 mmol) was dissolved in CH_2_Cl_2_ (8 mL). The solution was cooled to −78°C, and BCl_3_ (1 M in CH_2_Cl_2_, 1.2 mL) was added dropwise. The reaction mixture was stirred at −78°C and warmed to 0°C over 1.5 h. The reaction mixture was cooled to −78°C, and MeOH (0.5 mL) and aq. HCl (2 N, 5 mL) were added. The solution was diluted with EtOAc (150 mL), and washed with HCl (30 mL), 5% aq. NaHCO_3_ (30 mL), and brine (30 mL). After filtration and evaporation, the residue was purified on silica (step gradient: %0 then 5% isopropanol/EtOAc). The product **(1** ***S*** **,3** ***R*** **,4** ***R*** **)-12** was obtained as a colorless oil (31 mg, 80%). ^1^H NMR δ 8.20 (s, 1H), 7.59 (d, *J* = 7.5, 1H), 7.38 (t, *J* = 0.8, 1H) 7.22 (app. tt, *J* = 7.3, 1.1, 1H), 7.14 (app. tt, *J* = 7.3, 1.2, 1H), 7.06 (d, *J* = 2.1, 1H), 6.50 (d, *J* = 7.3, 1H), 5.75 (t, *J* = 5.6, 1H), 4.87 (m, 1H), 4.39 (d, *J* = 11.8, 1H), 4.14 (m, 1H), 3.81 (m, 1H), 3.55 (dd, *J* = 13.2, 6.4, 1H), 3.49 (dd, *J* = 12.8, 6.4, 1H), 3.04 (dt, *J* = 3.4, 11.6, 1H), 2.91 (dt, *J* = 0.9, 6.4, 2H), 2.52 (dt, *J* = 3.3, 11.6, 1H), 2.06 (m, 1H), 2.02 (s, 3H), 1.81 (m, 3H), 1.42 (m, 1H), 1.26 (m, 2H), 1.06 (m, 1H); ^13^C NMR (100 MHz) δ 210.4, 176.0, 170.0, 136.5, 127.3, 122.5, 122.4, 119.7, 118.8, 112.7, 111.4, 63.4, 59.5, 48.7, 45.9, 39.9, 30.4, 29.2, 25.68, 25.67, 25.2, 23.4; ESI^+^ HRMS m/z 400.2260 [M+H]^+^. Calculated for C_22_H_30_N_3_O_4_ 400.2236, By the same procedure, the minor isomer ***rac*** **-12** was obtained as an oil (20 mg, 80%). ^1^H NMR δ 8.39 (s, 1H), 7.56 (d, *J* = 7.9, 1H), 7.37 (d, *J* = 8.0, 1H), 7.25 (br s, 1H), 7.20 (dt, *J* = 1.1, 7.6, 1H), 7.12 (dt, *J* = 0.8, 7.5, 1H), 7.03 (s, 1H), 5.86 (t, *J* = 5.1, 1H), 4.53 (ddd, *J* = 2.7, 4.6, 7.4, 1H), 4.08 (br s, 1H), 3.93 (dd, *J* = 2.4, 11.8, 1H), 3.73 (dd, *J* = 4.4, 11.8, 1H), 3.53 (dd, *J* = 6.6, 13.2, 1H), 3.48 (dd, *J* = 6.6, 13.2, 1H), 3.00 (dt, *J* = 3.2, 11.3, 1H), 2.90 (t, *J* = 6.6, 2H), 2.48 (dt, *J* = 3.3, 11.7, 1H), 2.08 (s, 3H), 1.90 (d, *J* = 3.2, 1H), 1.76 (m, 3H), 1.40 (m, 1H), 1.21 (m, 2H), 1.09 (m, 1H); ^13^C NMR δ 213.9, 175.8, 170.9, 136.6, 127.3, 122.5, 122.4, 119.6, 118.7, 112.6, 111.5, 63.2, 60.3, 48.2, 47.3, 40.0, 30.3, 28.8, 25.6, 25.4, 25.2, 23.3; ESI^+^ HRMS m/z 422.2063 [M+Na]^+^. Calculated for C_22_H_29_N_3_O_4_·Na 422.2056.

#### Ac–Ser(PO(OBn)_2_)–Ψ[C = OCH]–Pip–2-(indol-3-yl)-ethylamine (1*S*,3*R*,4*R*)-13 and *rac*-13

To a solution of **(1** ***S*** **,3** ***R*** **,4** ***R*** **)-12** (33 mg, 0.083 mmol) in THF (10 mL) was added 5-ethylthio–1*H*–tetrazole (32 mg, 0.25 mmol) and *O,O*-dibenzyl-*N,N*-diethylphosphoramidite (0.087 mL, 0.25 mmol) at rt. The mixture was stirred at rt for 16 h. The mixture was cooled to −40°C, a solution of 5–6 M *tert*-butyl hydroperoxide in decane (61 µL, 0.33 mmol) was added dropwise, and the mixture was stirred at −40°C for 10 min, then at rt for 30 min. The reaction was cooled to −40°C and quenched with Na_2_S_2_O_3_. The mixture was diluted with EtOAc (80 mL), washed with HCl (20 mL), 5% NaHCO_3_ (aq, 20 mL), brine (20 mL), and dried over Na_2_SO_4_. The product was concentrated *in vacuo*, and purified by semi-preparative HPLC (10% CH_3_CN/H_2_O for 3 min, then 10% to 90% CH_3_CN/H_2_O gradient over 10 min) to give **(1** ***S*** **,3** ***R*** **,4** ***R*** **)-13**, ret. time 12.2 min, as a colorless oil (20 mg, 36%). ^1^H NMR (CD_3_OD) δ 7.53 (dt, *J* = 7.9, 1.0, 1H), 7.32 (m, 11H), 7.06 (ddd, *J* = 1.1, 7.0, 8.1, 1H), 7.01 (s, 1H), 6.97 (ddd, *J* = 0.9, 7.0, 8.0, 1H), 5.04 (d, *J* = 6.6, 2H), 5.02 (d, *J* = 6.1, 2H), 4.93 (dd, *J* = 3.8, 6.8, 1H), 4.53 (ddd, *J* = 3.9, 6.9, 10.8, 1H), 4.16 (ddd, *J* = 7.1, 8.2, 11.1, 1H), 3.37 (m, 2H), 2.95 (ddd, *J* = 3.2, 10.8, 12.2, 1H), 2.85 (m, 2H), 2.51 (m, 1H), 2.10 (m, 1H), 1.94 (s, 3H), 1.86 (m, 1H), 1.78 (m, 2H), 1.32 (m, 3H), 1.10 (m, 1H); ^13^C NMR δ 209.5, 177.2, 173.2, 138.1, 137.1, 137.0, 129.73, 129.68, 129.23, 129.19, 128.8, 123.5, 122.3, 119.6, 119.3, 113.2, 112.2, 71.0, 67.0 (d, ^3^ *J*_P-C_ = 5.0), 59.1 (d, ^2^ *J*_P-C_ = 7.5), 50.6, 47.3, 41.3, 31.1, 30.2, 26.6, 26.5, 26.3, 22.4; ^31^P NMR (202 MHz): δ −0.40; ESI^+^ HRMS m/z 660.2846 [M+H]^+^. Calculated for C_36_H_43_N_3_O_7_P 660.2839. By the same procedure, the minor isomer ***rac*** **-13**, ret. time 12.0 min, was obtained as an oil (17 mg, 37%). ^1^H NMR δ 8.49 (s, 1H), 7.54 (d, *J* = 7.7, 1H), 7.36 (d, *J* = 8.0, 1H), 7.33 (m, 10H), 7.18 (ddd, *J* = 1.1, 7.1, 8.1, 1H), 7.10 (ddd, *J* = 1.0, 7.0, 8.0, 1H), 6.98 (d, *J* = 2.2, 1H), 6.91 (d, *J* = 8.3, 1H), 5.50 (t, *J* = 5.8, 1H), 5.02 (m, 4H), 4.88 (ddt, *J* = 1.6, 4.2, 8.0, 1H), 4.32 (ddd, *J* = 4.1, 6.4, 10.5, 1H), 4.27 (ddd, *J* = 4.7, 6.3, 10.4, 1H) 3.58 (ddt, *J* = 6.0, 7.7, 13.5, 1H), 3.43 (ddt, *J* = 5.5, 6.5, 13.8, 1H), 2.95 (dt, *J* = 3.2, 11.2, 1H), 2.86 (m, 2H), 2.37 (dt, *J* = 3.5, 11.6, 1H), 1.94 (s, 3H), 1.84 (d, *J* = 11.8, 1H), 1.74 (m, 3H), 1.39 (m, 1H), 1.16 (m, 3H); ^13^C NMR δ 209.5, 174.7, 170.5, 136.6, 135.9, 135.88, 135.8, 128.74, 128.71, 128.69, 128.13, 128.11, 127.4, 122.5, 122.2, 119.5, 118.7, 112.6, 111.5, 69.60 (d, ^2^ *J*_P-C_ = 3.9), 69.55 (d, ^2^ *J*_P-C_ = 3.8), 65.4 (d, ^3^ *J*_P-C_ = 5.4), 57.1 (d, ^2^ *J*_P-C_ = 9.1), 47.4, 47.1, 39.5, 30.0, 28.7, 25.4, 25.3, 25.2, 23.2; ^31^P NMR: δ −0.53; ESI^+^ HRMS m/z 660.2842 [M+H]^+^. Calculated for C_36_H_43_N_3_O_7_P 660.2839.

#### Ac–Ser(PO(OH)_2_)–Ψ[C = OCH]–Pip–2-(indol-3-yl)-ethylamine 1 and *rac*-2

Dibenzyl phosphate **13** (14 mg, 0.021 mmol) and 10% Pd/C (7 mg) were dissolved in MeOH (8 mL). The reaction was stirred under H_2_ (1 atm) at rt for 2 h. The reaction mixture was filtered through Celite, and washed with MeOH. After evaporation, the residue was purified by semi-preparative HPLC (5% CH_3_CN/H_2_O for 3 min, then 5% to 30% CH_3_CN/H_2_O gradient over 10 min) to provide **1**, ret. time 8.2 min, as a white solid after lyophilization (8.0 mg, 78%). Anal. HPLC, 254 nm, (gradient: 5% B for 3 min, then 5–90% B over 6 min), 6.0 min, 99.9%; ^1^H NMR (CD_3_OD) δ 7.54 (d, *J* = 7.7, 1H), 7.32 (d, *J* = 8.2, 1H), 7.07 (m, 2H), 6.99 (ddd, *J* = 0.9, 7.0, 8.0, 1H), 4.92 (m, 1H), 4.35 (m, 1H), 4.18 (m, 1H), 3.40 (m, 2H), 3.04 (dt, *J* = 3.0, 11.4, 1H), 2.89 (m, 2H), 2.52 (ddd, *J* = 3.4, 10.5, 12.2, 1H), 2.22 (dd, *J* = 2.2, 13.2, 1H), 2.00 (s, 3H), 1.83 (m, 3H), 1.35 (m, 3H), 1.11 (m, 1H); ^13^C NMR (CD_3_OD) δ 209.8, 177.7, 173.2, 138.1, 128.8, 123.6, 122.2, 119.5, 119.3, 113.2, 112.2, 65.5, 59.2 (d, ^2^ *J*_P-C_ = 12), 50.4, 47.1, 41.2, 31.2, 30.3, 26.8, 26.6, 26.2, 22.3; ^31^P NMR (CD_3_OD) δ 1.44; ESI^+^ HRMS m/z 480.1906 [M+H]^+^. Calculated for C_22_H_31_N_3_O_7_P 480.1900. By the same procedure, the minor isomer ***rac-*** **2**, ret. time 8.0 min, was obtained as a white powder (5.5 mg, 70%). Anal. HPLC, 254 nm, (gradient: 5% B for 3 min, then 5–90% B over 6 min), 6.8 min, 99.1%; ^1^H NMR (dimethylsulfoxide (DMSO)-*d*_6_): δ 10.82 (br s, 1H), 8.25 (br s, 1H), 7.89 (t, *J* = 5.6, 1H), 7.50 (d, *J* = 7.8, 1H), 7.31 (d, *J* = 7.7, 1H), 7.10 (d, *J* = 1.5, 1H), 7.04 (t, *J* = 7.4, 1H), 6.96 (t, *J* = 7.2, 1H), 4.67 (m, 1H), 4.14 (m, 1H), 3.96 (m, 1H), 3.24 (m, 2H), 2.99 (t, *J* = 10.4, 1H), 2.73 (m, 2H), 2.36 (dt, *J* = 3.6, 11.3, 1H), 1.94 (m, 1H), 1.87 (s, 3H), 1.82 (m, 1H), 1.68 (m, 2H), 1.24 (m, 3H), 1.02 (m, 1H); ^13^C NMR (CD_3_OD): δ 210.5, 177.2, 173.1, 138.1, 128.8, 123.6, 122.2, 119.5, 119.3, 113.3, 112.2, 65.4, 60.0, 49.4, 49.3, 41.2, 31.3, 29.3, 26.5, 26.4, 26.2, 22.6; ^31^P NMR (DMSO-*d*_6_): δ −2.93; ESI^+^ MS m/z 480.18 [M+H]^+^. Calculated for C_22_H_31_N_3_O_7_P 480.19.

### X-ray structures

Crystal structure **(1** ***S*** **,3** ***R*** **,4** ***R*** **)-11**: Colorless needles (0.31×0.02×0.004 mm^3^) were recrystallized from EtOAc∶hexanes (1∶2) at rt. The chosen crystal was centered on the goniometer of an Oxford Diffraction Nova diffractometer operating with CuKα radiation. The data collection routine, unit cell refinement, and data processing were carried out with the program CrysAlis [40]. The Laue symmetry and systematic absences were consistent with the monoclinic space groups *P*2_1_ and *P*2_1_/*m*. Since the molecule was known to be enantiomerically pure, the chiral space group, *P*2_1_, was chosen. The structure was solved by direct methods and refined using SHELXTL NT [41]. The asymmetric unit of the structure comprises one crystallographically independent molecule. The final refinement model involved anisotropic displacement parameters for non-hydrogen atoms and a riding model for all hydrogen atoms. Since there were no heavy atoms, the absolute configuration could not be determined from the Friedel pairs; the Friedel pairs were therefore merged for the final refinement. The absolute configuration was assigned by reference to C(19) of known S-configuration. Relative to C(19), C(17) and C(12) are both R-configuration (Figure 4). SHELXTL NT was used for molecular graphics generation [41]. Deposited Cambridge Crystallographic Data Centre (CCDC) 782064. Crystal structure ***rac*** **-11**: Colorless plates (0.004×0.06×0.12 mm^3^) were recrystallized from EtOAc∶hexanes (1∶2) at rt. Data were collected as for **(1** ***S*** **,3** ***R*** **,4** ***R*** **)-11** above. The Laue symmetry and systematic absences were consistent with the monoclinic space group *P*2_1_/*c*. The structure was solved as for **(1** ***S*** **,3** ***R*** **,4** ***R*** **)-11**. The asymmetric unit of the structure comprised one crystallographically independent molecule. The final refinement model involved anisotropic displacement parameters for non-hydrogen atoms, and a riding model for all hydrogen atoms. The benzyl group was modeled with positional disorder, with the two positions refining to relative occupancies of 52.9(3)% and 47.1(3)% (Figure 4). Deposited CCDC 782063.

### Pin1 Enzyme Assays

The Pin1 inhibition assay was performed at 4°C in 35 mM 4-(2-hydroxyethyl)piperazine-1-ethanesulfonic acid (HEPES) pH 7.8 in a total assay volume of 1.2 mL as published [10]. Inhibitors were dissolved in DMSO∶H_2_O (2∶1) and 20 µL of stock was added to give final concentrations of **1**: 12, 50, 100, 200, 400, 810 µM, and ***rac*** **-2**: 10, 20, 40, 60, 120, 240, 480 µM, pre-equilibrated with Pin1 in HEPES at 4°C for 15 min. The Pin1 final concentration in the assay was 67 nM. The final concentration of succinyl–Ala–Glu–*cis*-Pro–Phe-*p*-nitroanilide was 34 µM. For each concentration, the assay was performed in duplicate. The plot of % Inhibition vs. log [I] (µM) produced sigmoidal curves by fitting all of the experimental data to Eq. 1 using TableCurve v3 for win32 (Dataset S2). The IC_50_ values were derived from the fitted equation at 50% inhibition of enzyme activity (Eq. 1), where a, b, c, and d are fitted constants given on the plots for compounds **1** and ***rac*** **-2** (Dataset S2).
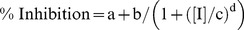
(1)


### Computational Methods

Models of three stereoisomeric ketones were based on the X-ray structure of peptide inhibitor, Ac–Phe–pThr–Pip–Nal–Gln–NH_2_, bound to Pin1, protein data bank (PDB) 2Q5A, using Sybyl 8.1.1 (Figure 5) [32]. In each case, the Pip nitrogen was changed to a CH group with the appropriate stereochemistry. The naphthyl (Nal) side chains were modified to indoles, and the Nal carbonyls were deleted. The Thr methyl groups, the Gln, and all except the alpha-carbon and carbonyl of the Phe residues (which became acetyl groups) were deleted. Further modification of the starting structures included drafting a diequatorial chair conformation for the cyclohexyl rings, inversion of the Ser stereochemistry for **(1** ***R,*** **3** ***R,*** **4** ***R*** **)-2**, inversion of the cyclohexyl ring stereocenters for **(1** ***S,*** **3** ***S,*** **4** ***S*** **)-2**, and manual rotation of torsions of **(1** ***R,*** **3** ***R,*** **4** ***R*** **)-2** to bring the phosphate and indole groups close to these groups in the original crystal structure. Explicit waters from the crystal structure were retained. Protein termini charges, all hydrogens, and Amber FF02 atom types were added manually to the inhibitor atoms, phosphate groups, and Arg guanidines. The 3 oxygens of the phosphate groups were given formal charges of −0.67 prior to computation of Gasteiger-Marsili charges. Energy minimization, with geometry optimization of the inhibitors and all Pin1 residues within 8 Å of the inhibitors, was performed using Sybyl 8.1.1 with Gasteiger-Marsili charges, Amber FF02 force field, Powell conjugate gradient, gradient termination at 0.1 kcal/mol-Å, 8 Å non-bonded cut-off, and a dielectric constant of 1.0. Typically, gradient convergence was reached within 3000 iterations. Distances and angles were measured using Sybyl 8.1.1 [42].

Cyclohexane-1,2-*cis*-dial, cyclohexane-1,2-*trans*-dial, Ac–*cis*-Pro–OH, and Ac-Pro-OH with fixed *trans*-pyrrolidine (ω = −60°) conformation were geometry optimized using WebMO with Moller-Plesset 2, 6-31G(d), polarizable continuum model, and water as solvent [43]. For the twisted-amide conformation, the *trans*-pyrrolidine torsion angle was fixed to −155.6°, the angle found at the B3LYP STO-3G level of theory.

## Supporting Information

Dataset S1 HPLC chromatograms for **1** and ***rac*** **-2**. ^1^H, ^13^C, and ^31^P NMR spectra for compounds **1**, ***rac*** **-2**, and **7**–**13**.
(PDF)

Dataset S2 Pin1 inhibition plots for **1** and ***rac*** **-2**. Crystallographic data, CCDC 782064 and 782063 for **(1** ***S*** **,3** ***R*** **,4** ***R*** **)-11** and ***rac*** **-11** respectively, can be obtained free of charge from The Cambridge Crystallographic Data Centre via www.ccdc.cam.ac.uk/data_request/cif.
(PDF)
