# Supplementary material for: Cyclohexyl Ketone Inhibitors of Pin1 Dock in a Trans-Diaxial Cyclohexane Conformation
Source: PLoS One. 2012 Sep 19;7(9):e44226. doi: 10.1371/journal.pone.0044226 (PMC3446931; doi:10.1371/journal.pone.0044226)
Supplement: Dataset S1 — HPLC chromatograms for 1 and rac -2. 1H, 13C, and 31P NMR spectra for compounds 1, rac -2, and 7–13. (PDF) [file pone.0044226.s001.pdf]

Supporting Information

Cyclohexyl ketone inhibitors of Pin1 dock in a *trans*-diaxial cyclohexane conformation

Guoyan G. Xu, Carla Slebodnick, and Felicia A. Etzkorn*

Department of Chemistry, Virginia Tech, Blacksburg, VA 24061-0212, USA

Dataset S1. NMR Spectra and chromatograms for compounds **1**, ***rac*-2**, and **7 – 13**.

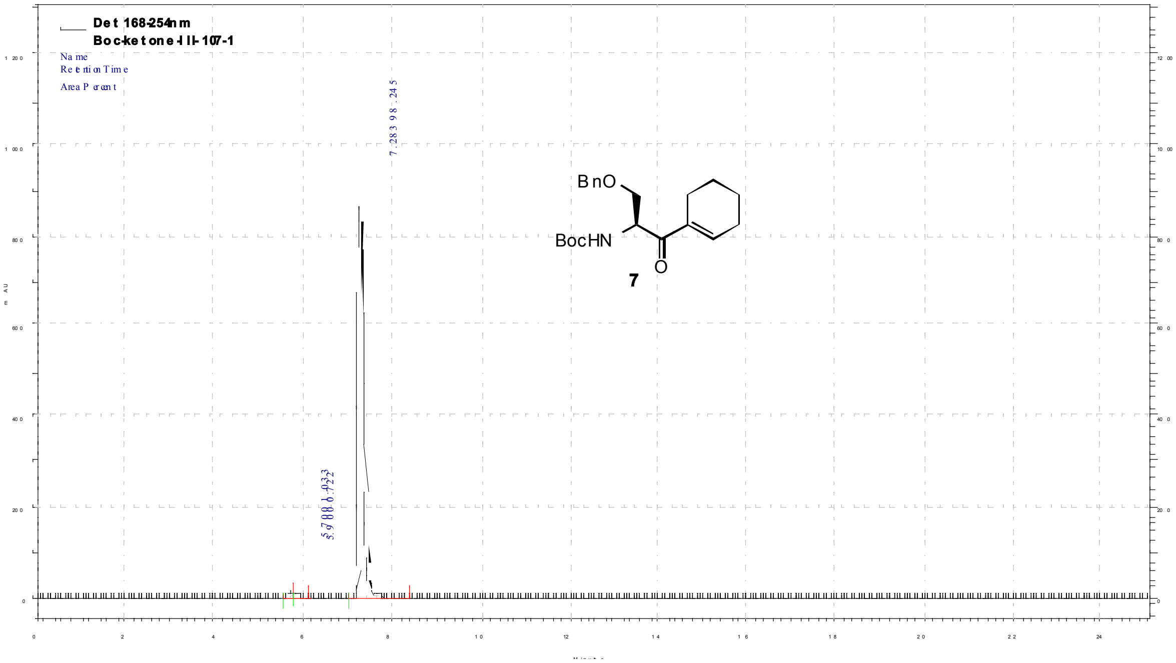

* Corresponding author. Phone: +1 540 231 2235; fax: +1 540 231 3255; e-mail: fetzkorn@vt.edu

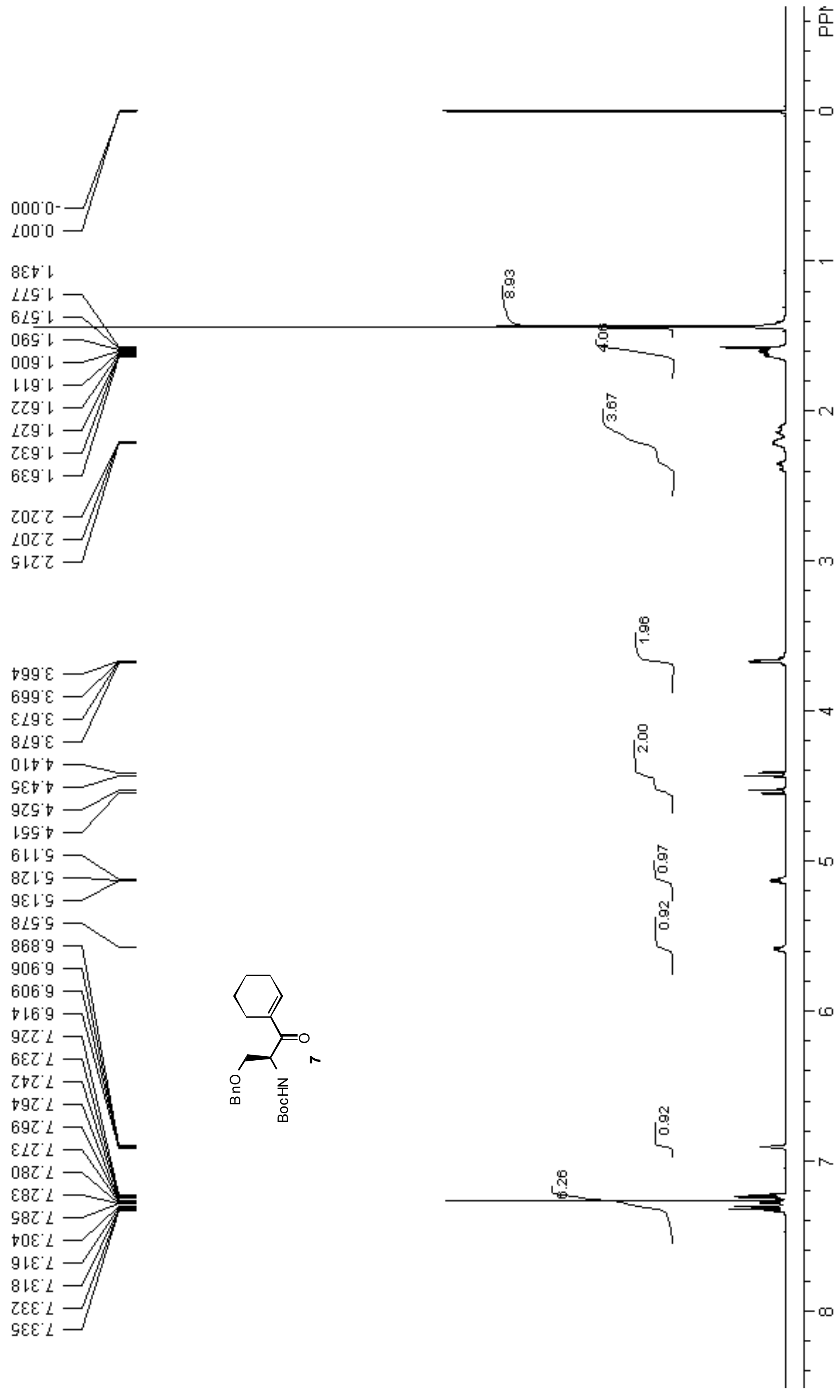
BnO
BocHN
O
7
-0.000
0.007
1.438
1.577
1.579
1.590
1.600
1.611
1.622
1.627
1.632
1.639
2.202
2.207
2.215
3.664
3.669
3.673
3.678
4.410
4.435
4.526
4.551
5.119
5.128
5.136
5.578
6.898
6.906
6.909
6.914
7.226
7.239
7.242
7.264
7.269
7.273
7.280
7.283
7.285
7.304
7.316
7.318
7.332
7.335
8.93
4.06
3.67
1.96
2.00
0.97
0.92
0.92
6.26
PPM

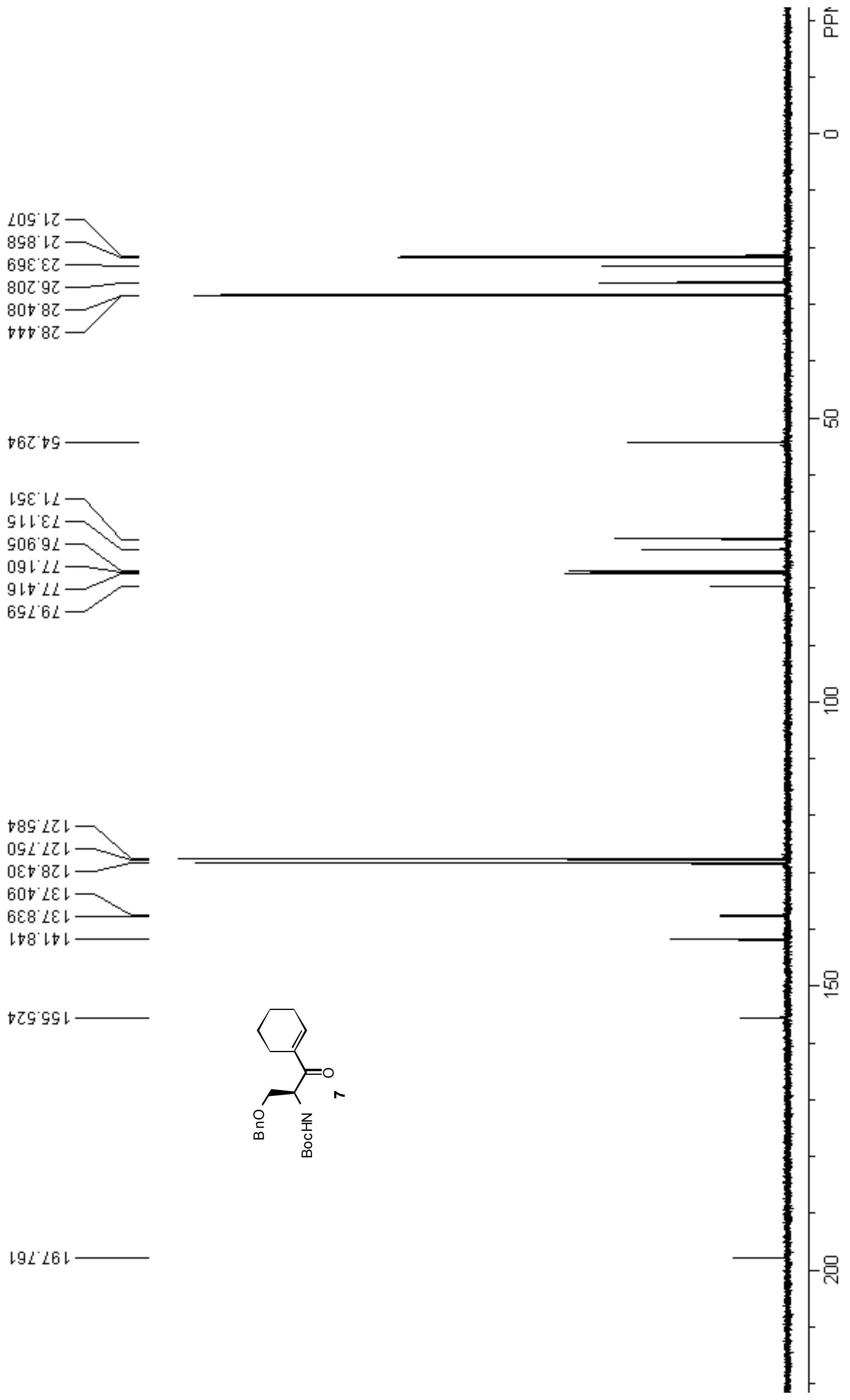
21.507
21.858
23.369
26.208
28.408
28.444
54.294
71.351
73.115
76.905
77.160
77.416
79.759
127.584
127.750
128.430
137.409
137.839
141.841
155.524
197.761
BnO
BocHN
O
7
PPM
0
50
100
150
200

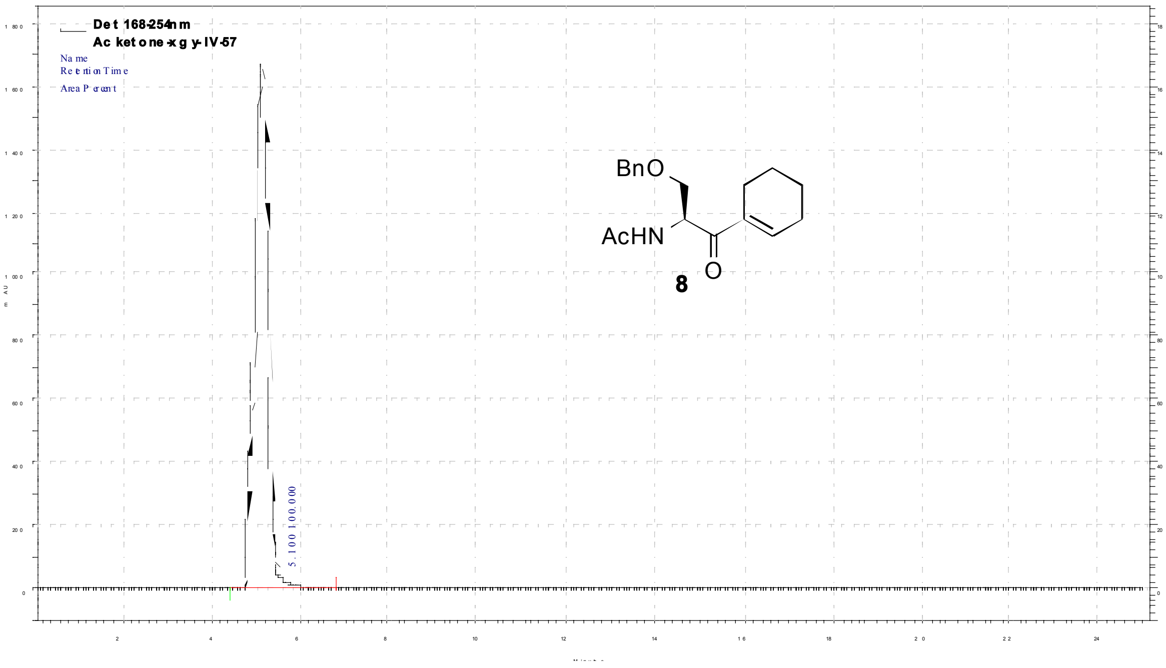
Det 168-254nm
Ac ketone-xgy-IV-57
Name
Retention Time
Area Percent
5.100 100.000
BnO
AcHN
O
8
mAU

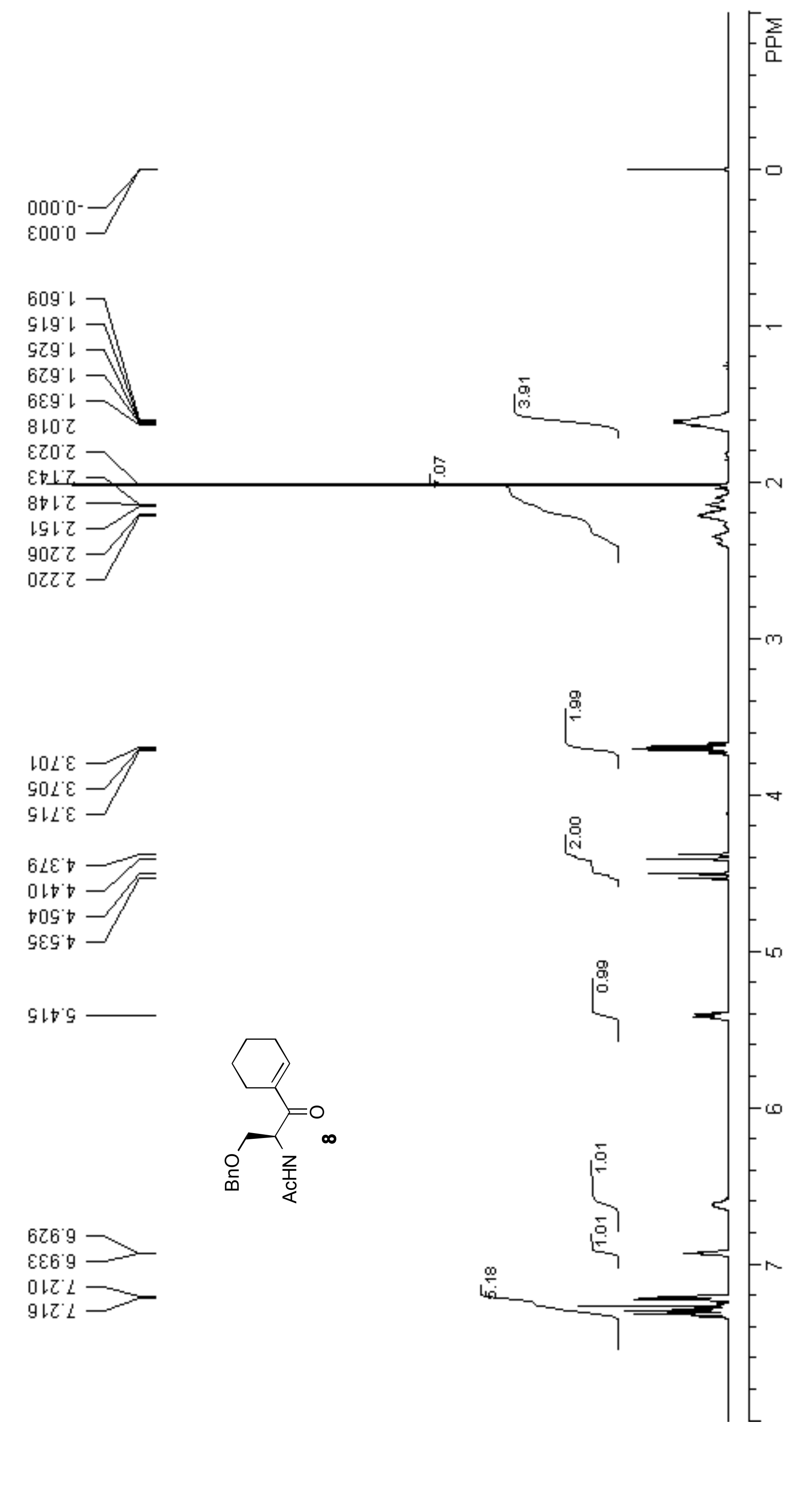
-0.000
0.003
1.609
1.615
1.625
1.629
1.639
2.018
2.023
2.143
2.148
2.151
2.206
2.220
3.701
3.705
3.715
4.379
4.410
4.504
4.535
5.415
6.929
6.933
7.210
7.216
3.91
7.07
1.99
2.00
0.99
1.01
1.01
5.18
BnO
AcHN
O
PPM
0
1
2
3
4
5
6
7

8

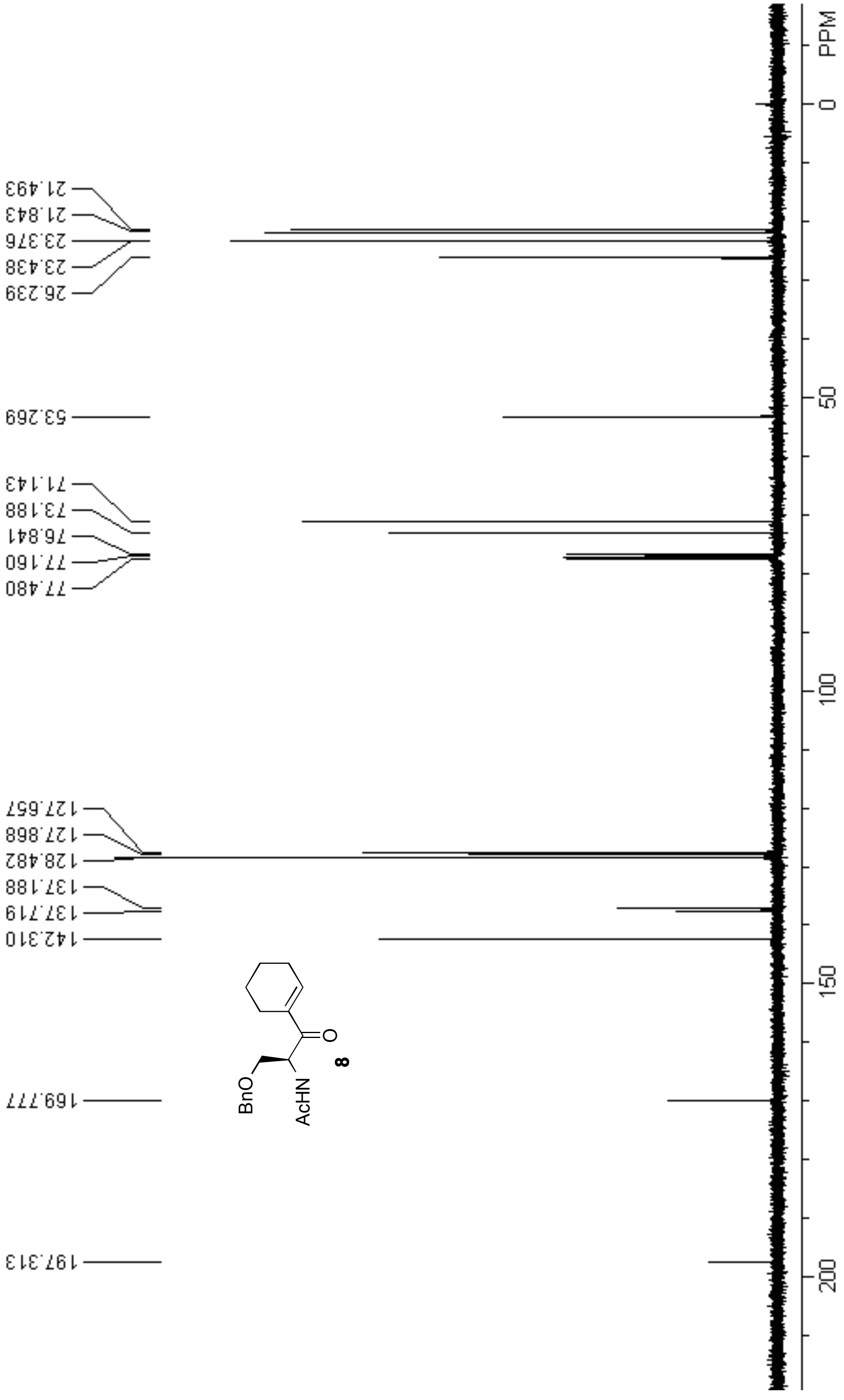
21.493
21.843
23.376
23.438
26.239
53.269
71.143
73.188
76.841
77.160
77.480
127.657
127.868
128.482
137.188
137.719
142.310
169.777
197.313
BnO
AcHN
O
8
PPM
0
50
100
150
200

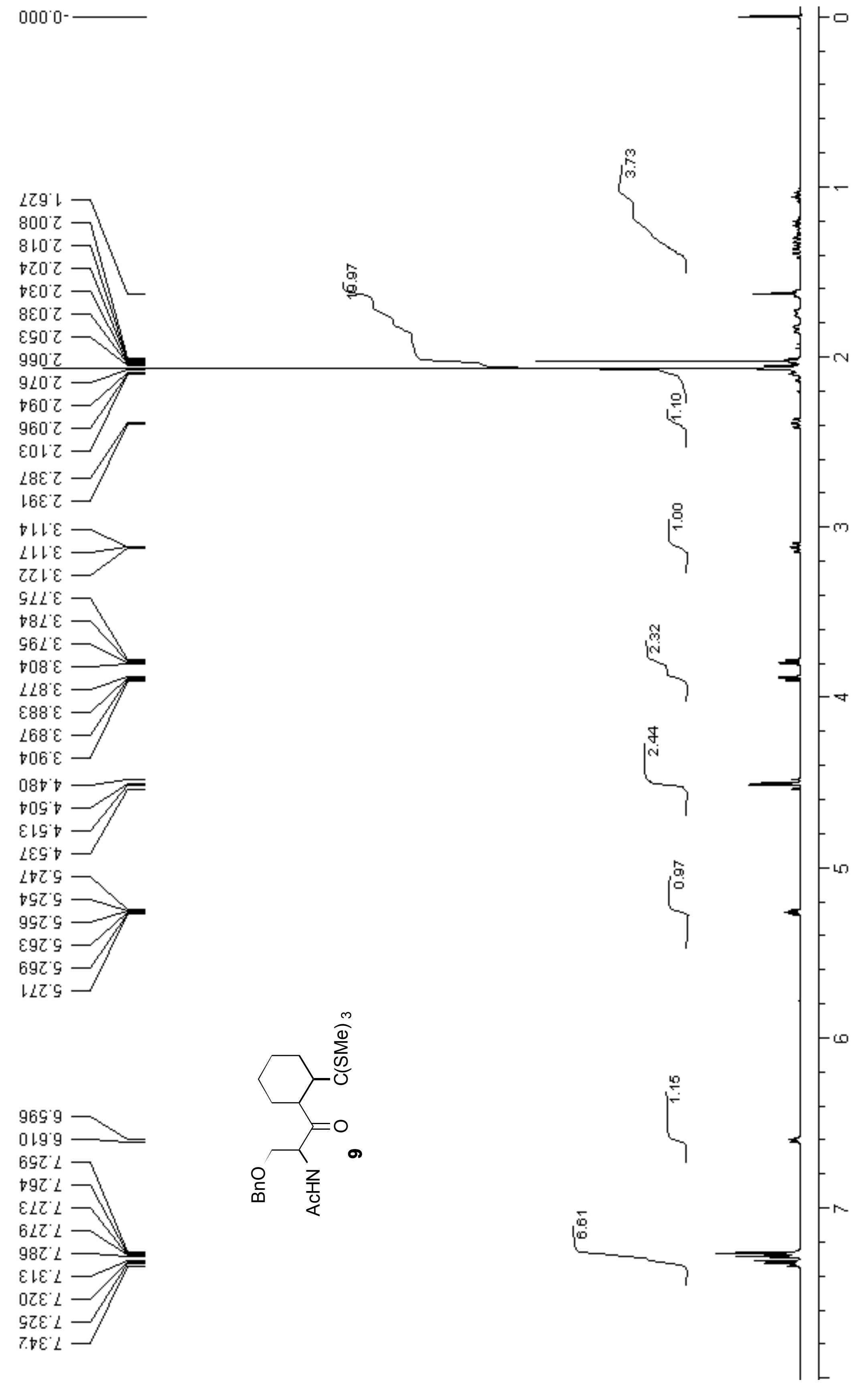

-0.000
1.627
2.008
2.018
2.024
2.034
2.038
2.053
2.066
2.076
2.094
2.096
2.103
2.387
2.391
3.114
3.117
3.122
3.775
3.784
3.795
3.804
3.877
3.883
3.897
3.904
4.480
4.504
4.513
4.537
5.247
5.254
5.256
5.263
5.269
5.271
6.596
6.610
7.259
7.264
7.273
7.279
7.286
7.313
7.320
7.325
7.342
BnO
AcHN
O
C(SMe)3
9
3.73
1.10
1.00
2.32
2.44
0.97
1.15
6.61
0
1
2
3
4
5
6
7

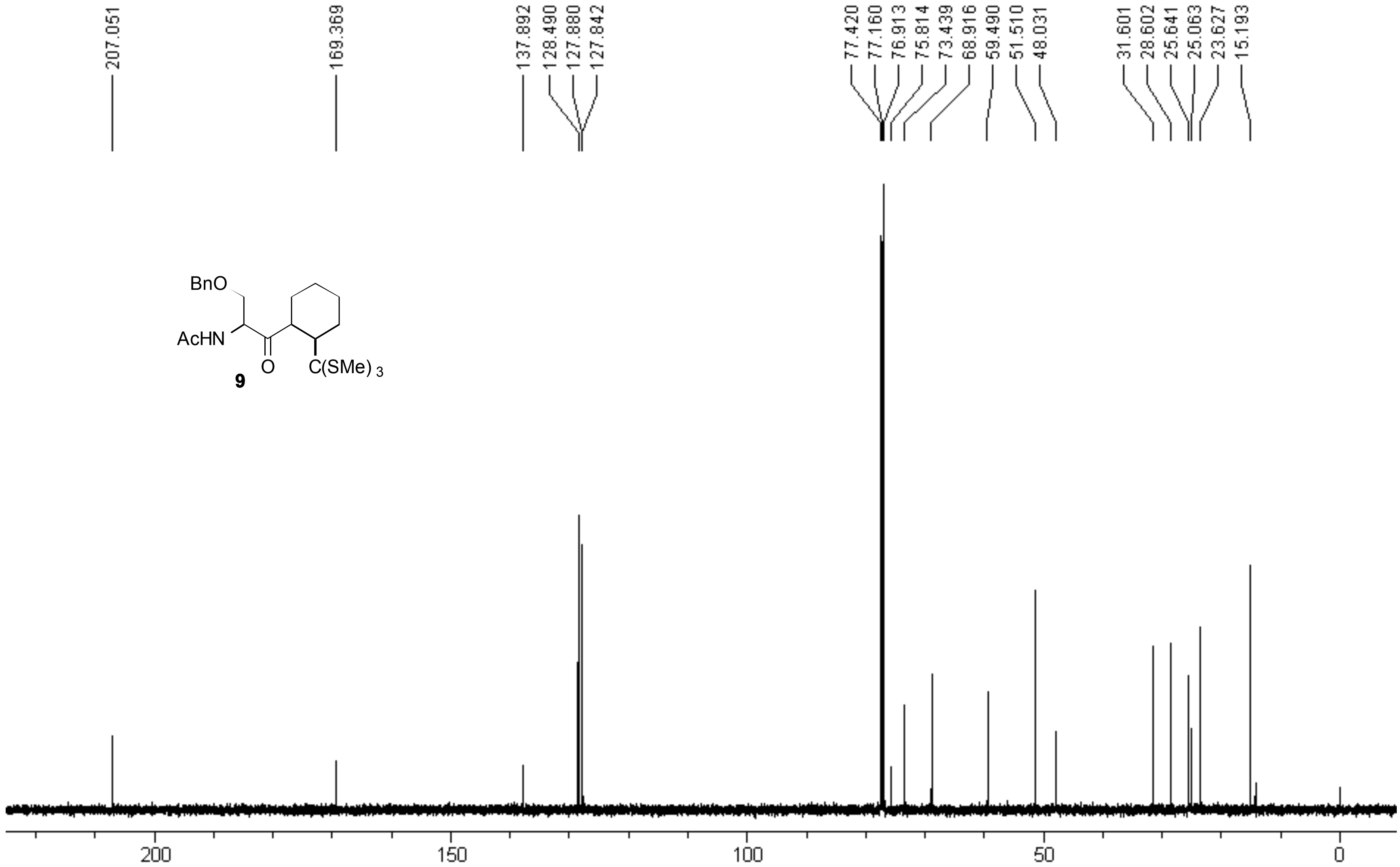
207.051
169.369
137.892
128.490
127.880
127.842
77.420
77.160
76.913
75.814
73.439
68.916
59.490
51.510
48.031
31.601
28.602
25.641
25.063
23.627
15.193
BnO
AcHN
O
C(SMe)3
9
200
150
100
50
0

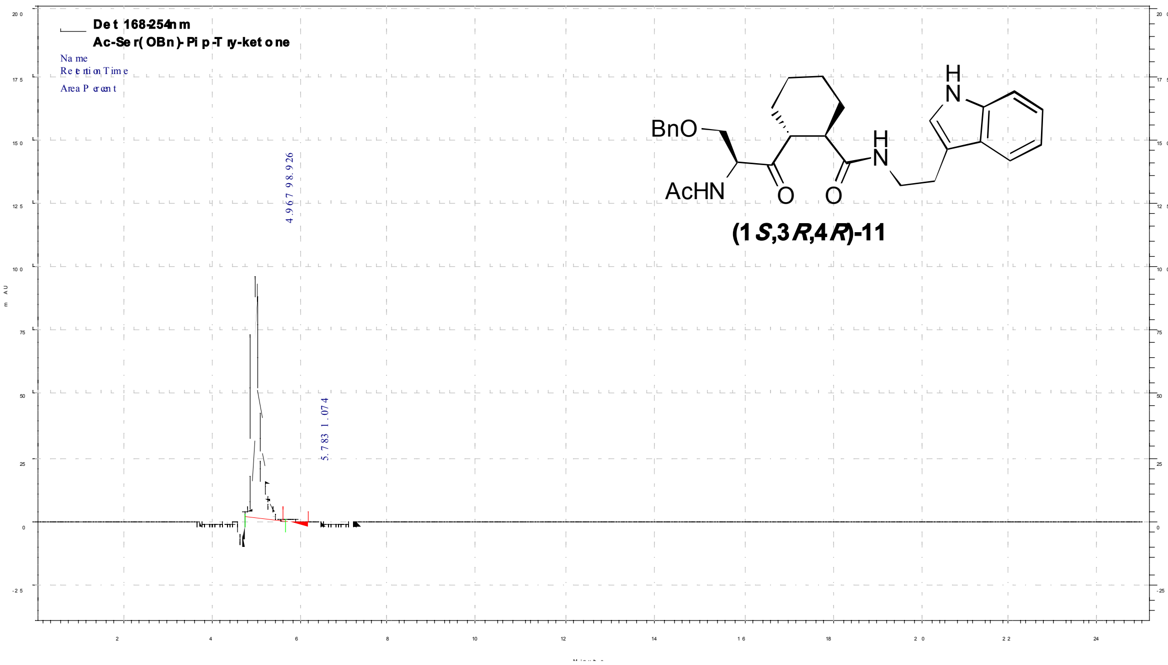
Det 168-254nm
Ac-Ser(OBn)-Pip-Try-ketone
Name
Retention Time
Area Percent
4.967 98.926
5.783 1.074
mAU
BnO
AcHN
O
O
H
N
H
N
(1S,3R,4R)-11

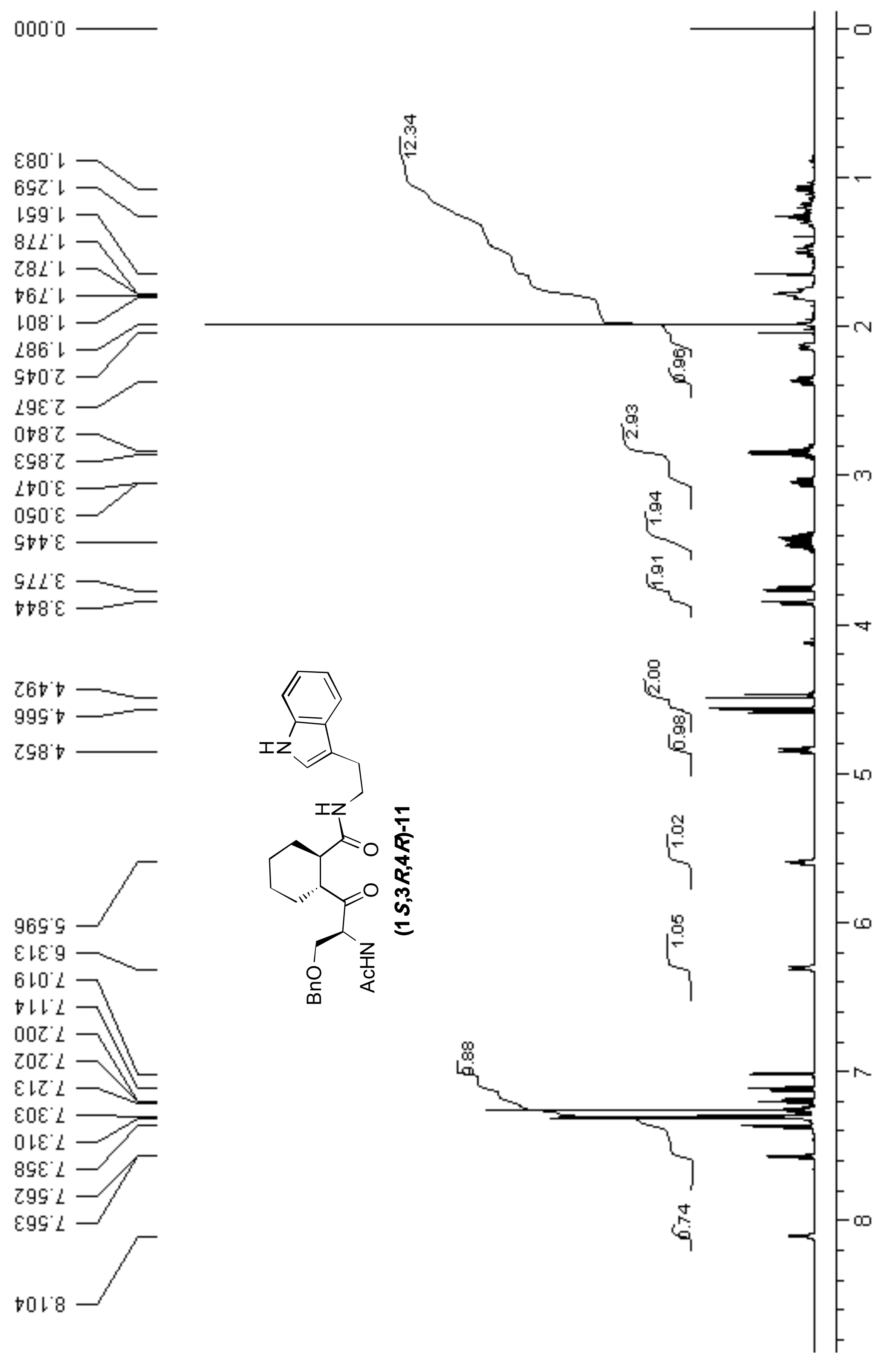
0.000
1.083
1.259
1.651
1.778
1.782
1.794
1.801
1.987
2.045
2.367
2.840
2.853
3.047
3.050
3.445
3.775
3.844
4.492
4.566
4.852
5.596
6.313
7.019
7.114
7.200
7.202
7.213
7.303
7.310
7.358
7.562
7.563
8.104
12.34
0.96
2.93
1.94
1.91
2.00
0.98
1.02
1.05
9.88
0.74
BnO
AcHN
O
O
NH
NH
(1S,3R,4R)-11

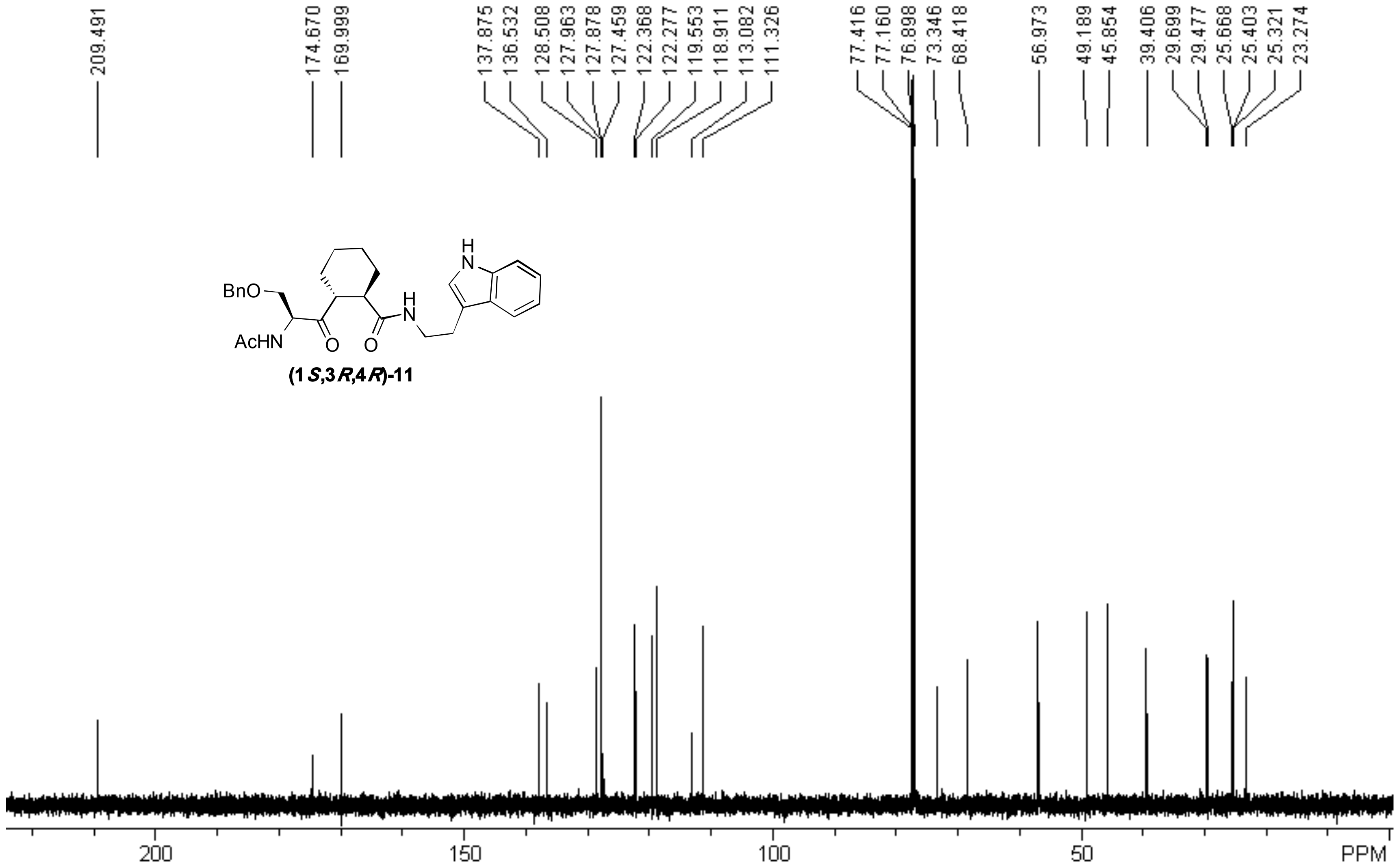

209.491
174.670
169.999
137.875
136.532
128.508
127.963
127.878
127.459
122.368
122.277
119.553
118.911
113.082
111.326
77.416
77.160
76.898
73.346
68.418
56.973
49.189
45.854
39.406
29.699
29.477
25.668
25.403
25.321
23.274
BnO
AcHN
(1*S*,3*R*,4*R*)-11
200
150
100
50
PPM

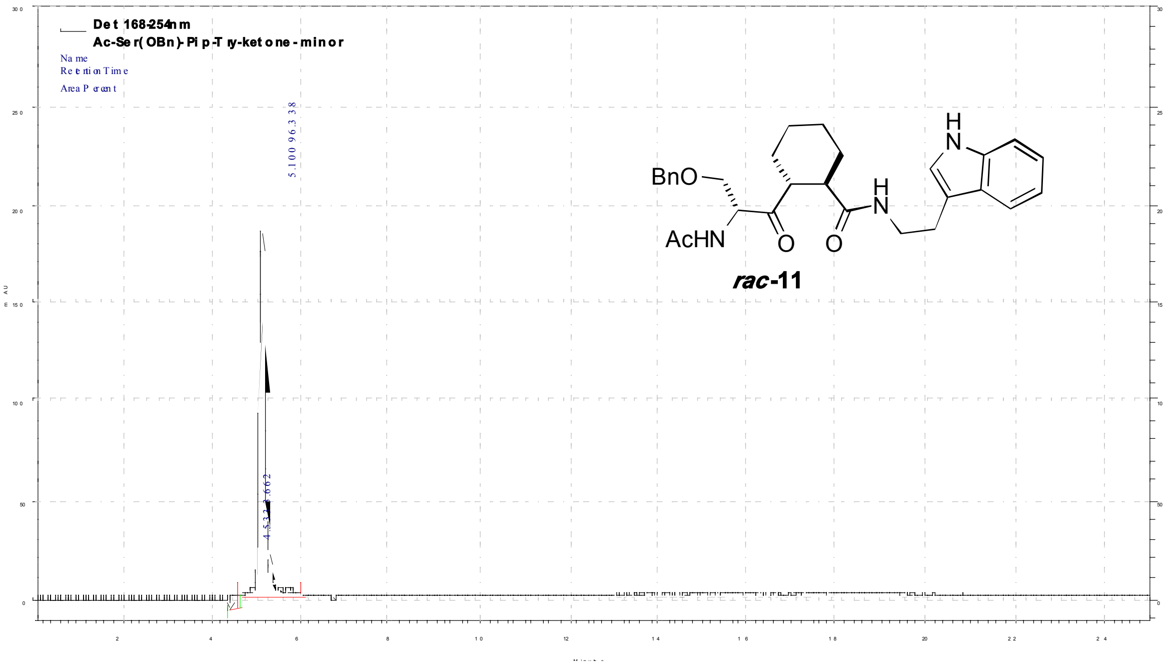

Det 168-254nm
Ac-Ser(OBn)-Pip-Try-ketone-minor
BnO
AcHN
O
O
H
N
H
N
rac-11

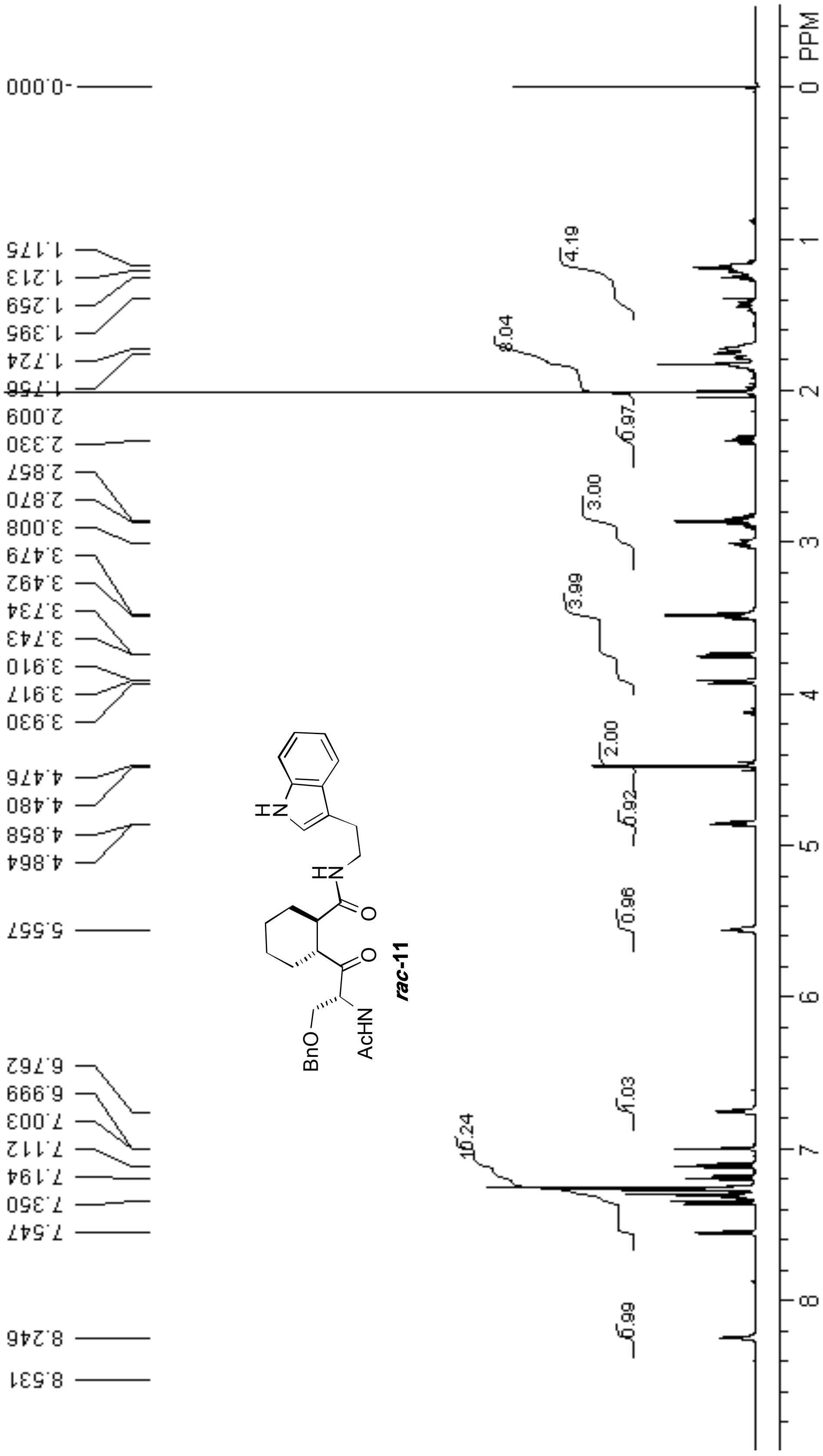
-0.000
1.175
1.213
1.259
1.395
1.724
1.756
2.009
2.330
2.857
2.870
3.008
3.479
3.492
3.734
3.743
3.910
3.917
3.930
4.476
4.480
4.858
4.864
5.557
6.762
6.999
7.003
7.112
7.194
7.350
7.547
8.246
8.531
4.19
8.04
0.97
3.00
3.99
2.00
0.92
0.96
1.03
10.24
0.99
BnO
AcHN
NH
O
rac-11
PPM
0
1
2
3
4
5
6
7
8

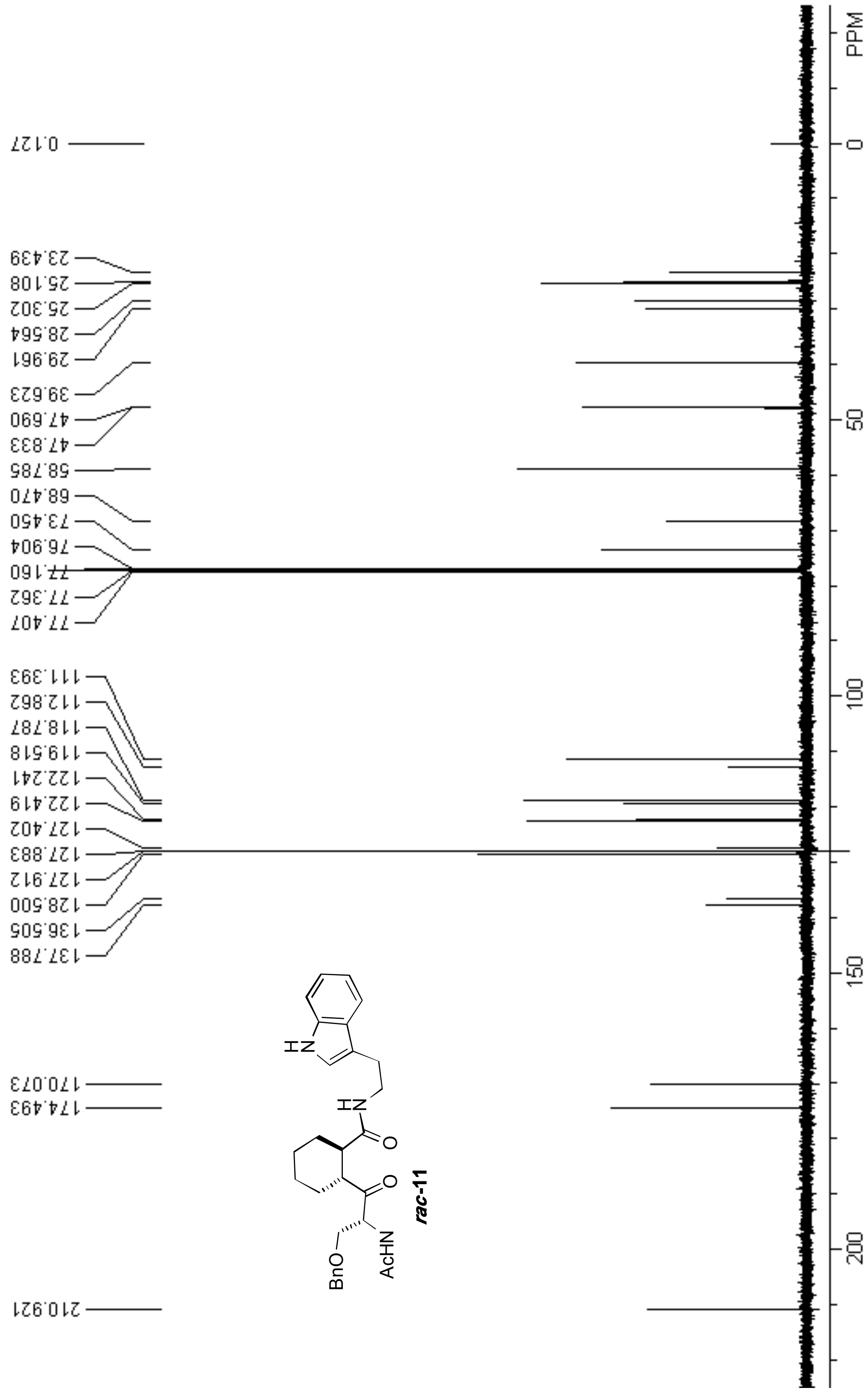
0.127
23.439
25.108
25.302
28.564
29.961
39.623
47.690
47.833
58.785
68.470
73.450
76.904
77.160
77.362
77.407
111.393
112.862
118.787
119.518
122.241
122.419
127.402
127.883
127.912
128.500
136.505
137.788
170.073
174.493
210.921
PPM
0
50
100
150
200
BnO
AcHN
O
HN
NH
***rac*-11**

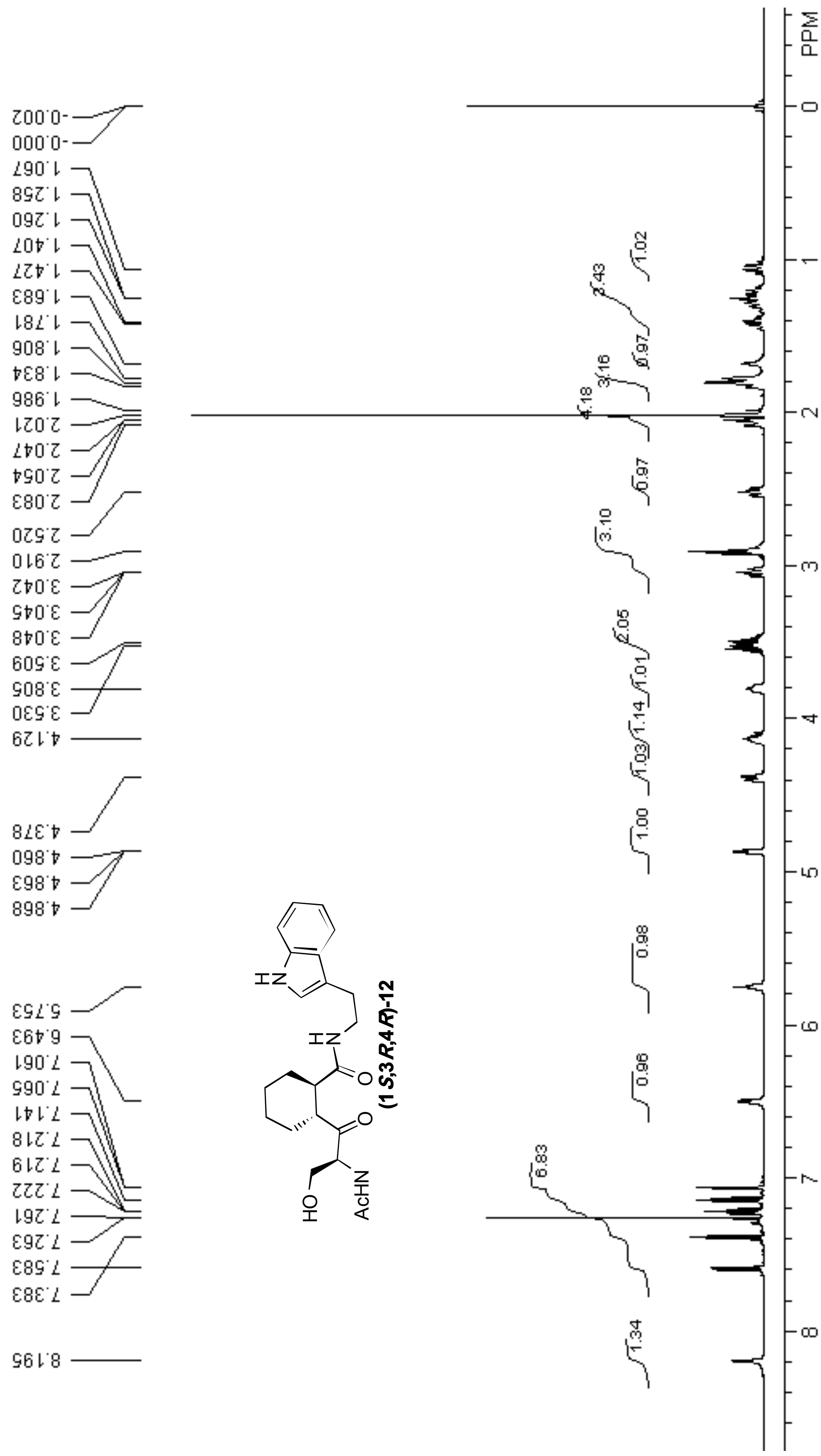

(1S,3R,4R)-12
AcHN
HO
PPM

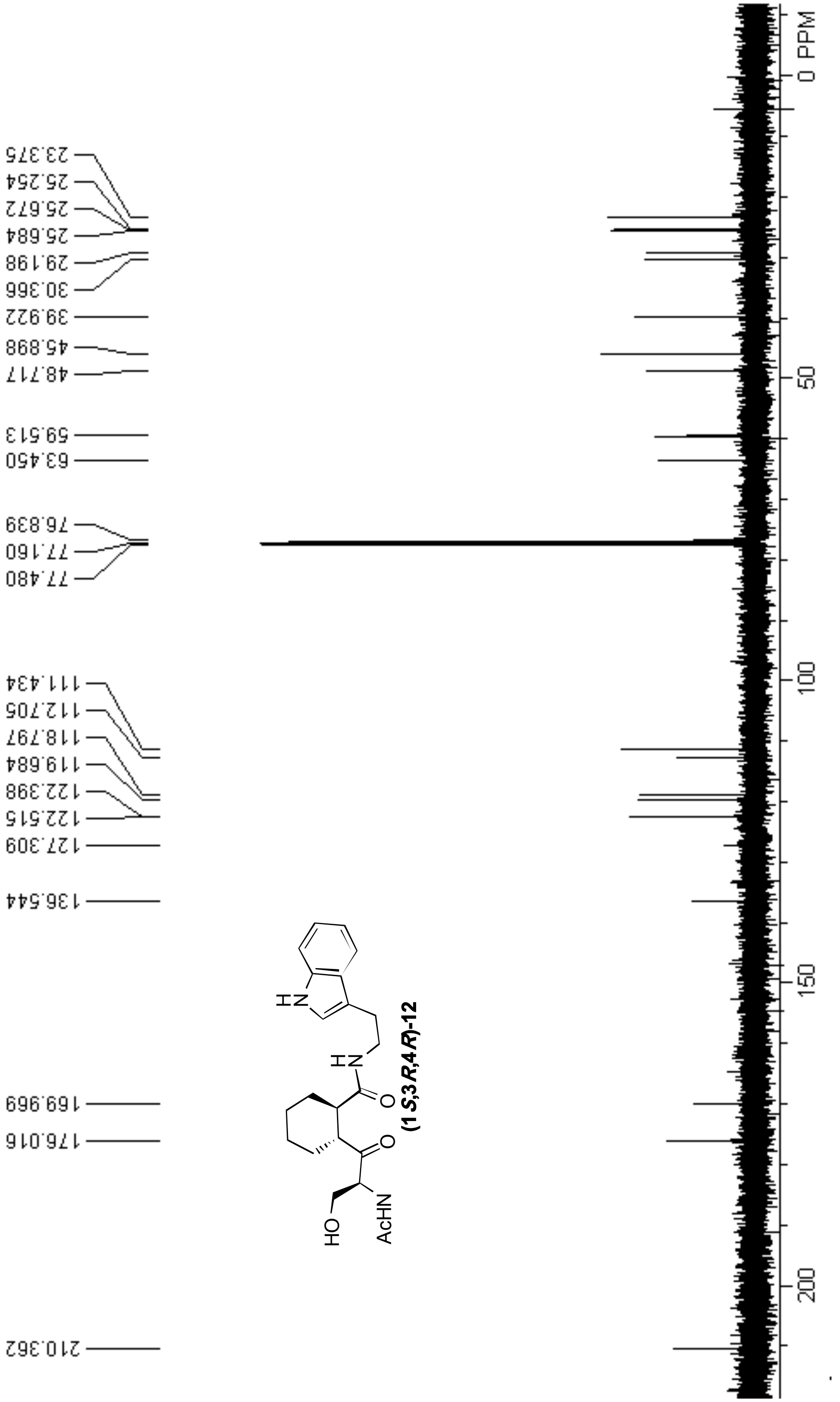
23.375
25.254
25.672
25.684
29.198
30.366
39.922
45.898
48.717
59.513
63.450
76.839
77.160
77.480
111.434
112.705
118.797
119.684
122.398
122.515
127.309
136.544
169.969
176.016
210.362
0 PPM
50
100
150
200
HO
AcHN
NH
O

(1*S*,3*R*,4*R*)-12

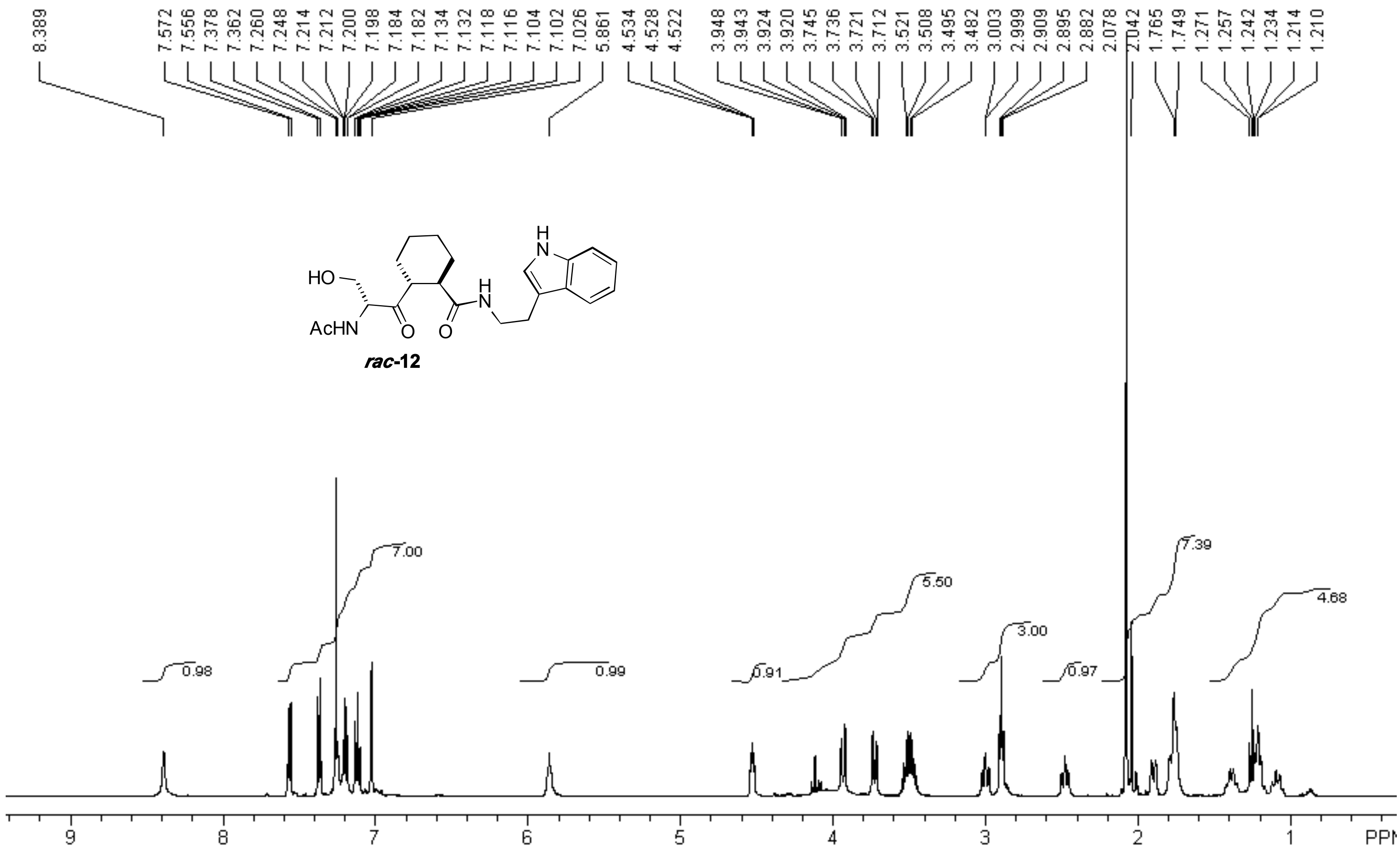
8.389
7.572
7.556
7.378
7.362
7.260
7.248
7.214
7.212
7.200
7.198
7.184
7.182
7.134
7.132
7.118
7.116
7.104
7.102
7.026
5.861
4.534
4.528
4.522
3.948
3.943
3.924
3.920
3.745
3.736
3.721
3.712
3.521
3.508
3.495
3.482
3.003
2.999
2.909
2.895
2.882
2.078
2.042
1.765
1.749
1.271
1.257
1.242
1.234
1.214
1.210
HO
AcHN
O
O
H
N
H
N
rac-12
7.00
5.50
3.00
7.39
4.68
0.98
0.99
0.91
0.97
9
8
7
6
5
4
3
2
1
PPM

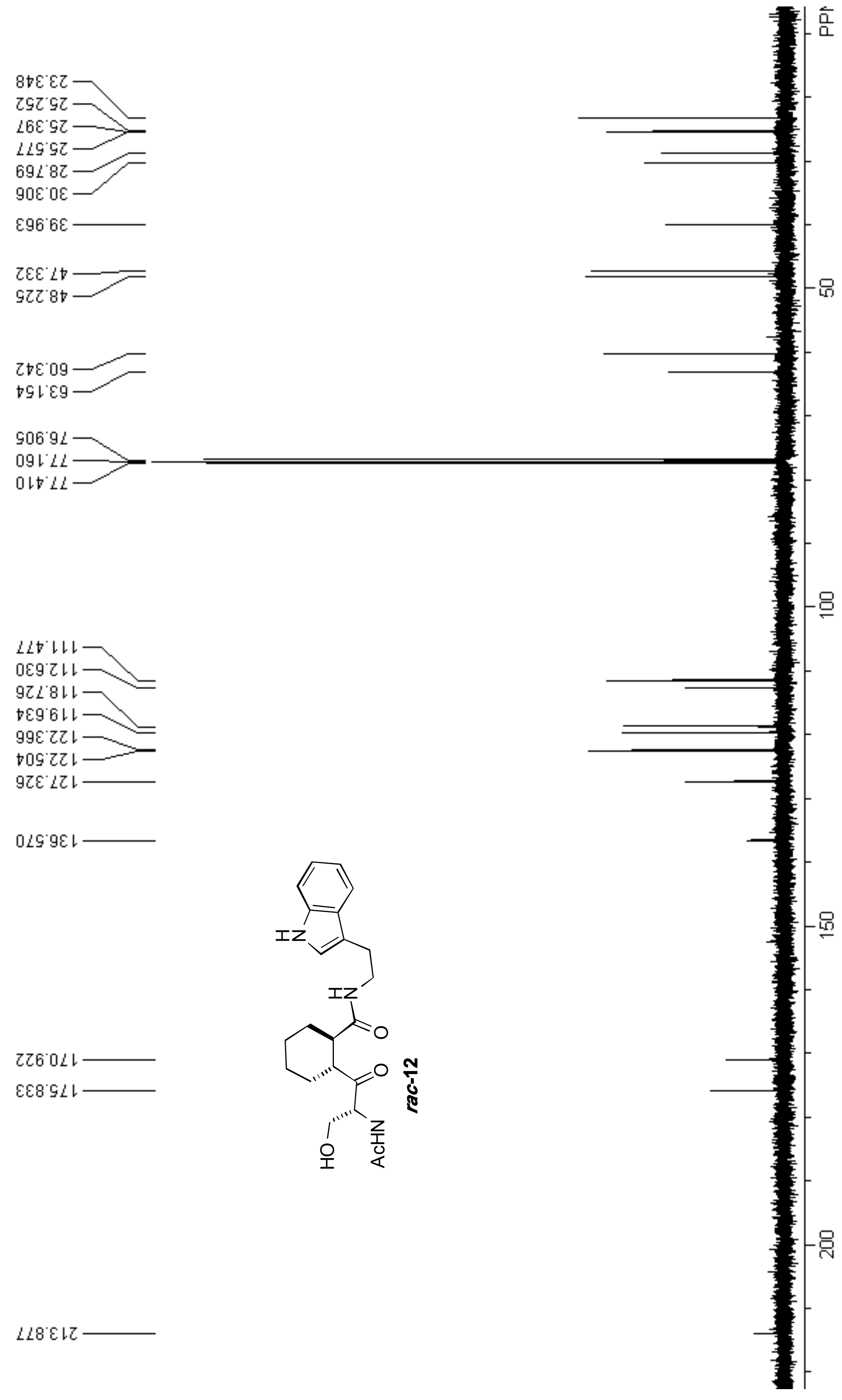
23.348
25.252
25.397
25.577
28.769
30.306
39.963
47.332
48.225
60.342
63.154
76.905
77.160
77.410
111.477
112.630
118.726
119.634
122.366
122.504
127.326
136.570
170.922
175.833
213.877
HO
AcHN
rac-12
PPM
50
100
150
200

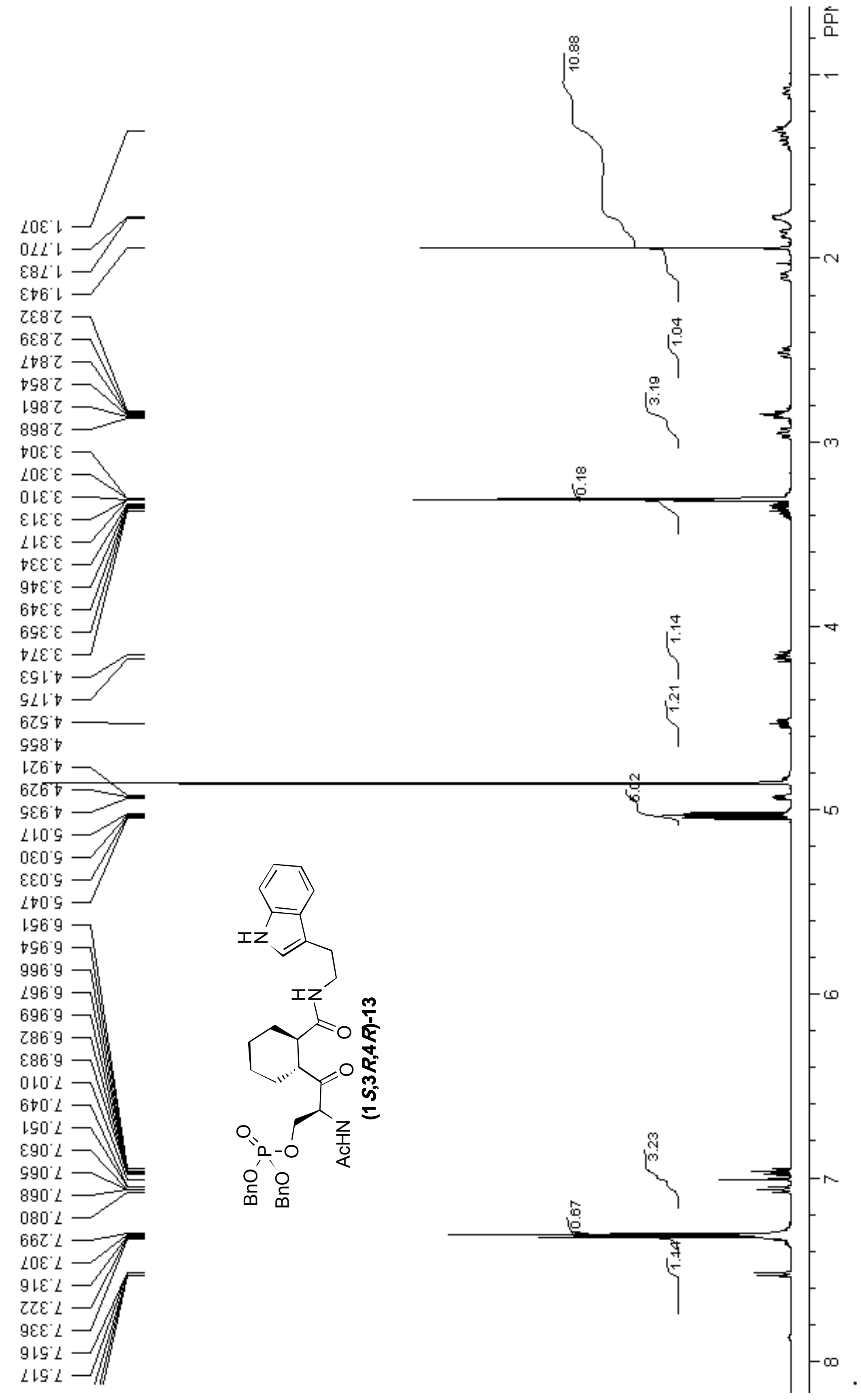
1.307
1.770
1.783
1.943
2.832
2.839
2.847
2.854
2.861
2.868
3.304
3.307
3.310
3.313
3.317
3.334
3.346
3.349
3.359
3.374
4.153
4.175
4.529
4.855
4.921
4.929
4.935
5.017
5.030
5.033
5.047
6.951
6.954
6.966
6.967
6.969
6.982
6.983
7.010
7.049
7.051
7.063
7.065
7.068
7.080
7.299
7.307
7.316
7.322
7.336
7.516
7.517
BnO
BnO
AcHN
10.88
1.04
3.19
0.18
1.14
1.21
6.02
3.23
0.67
1.44
PPM

(1*S*,3*R*,4*R*)-13

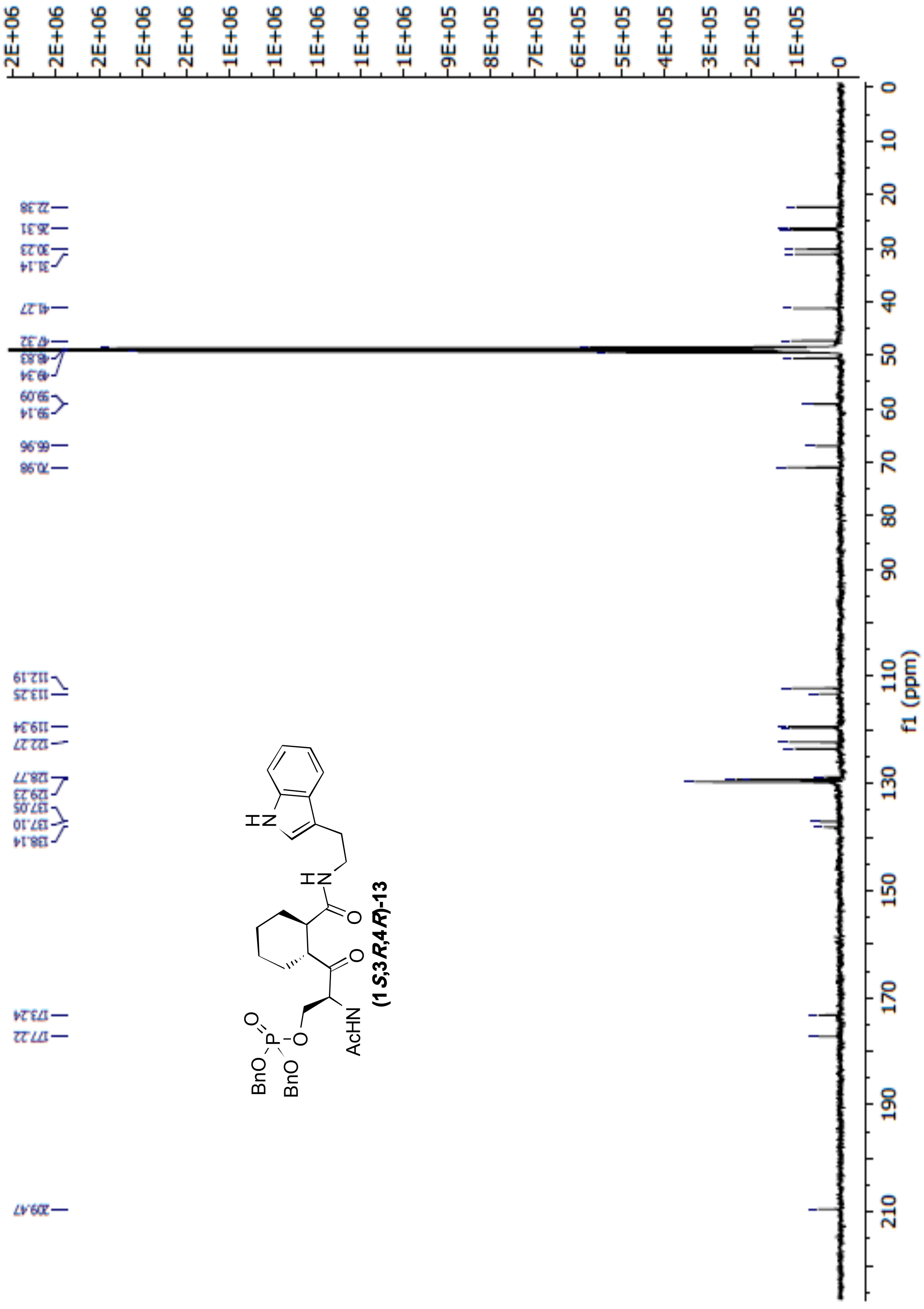
(1S,3R,4R)-13
BnO
BnO
AcHN
f1 (ppm)

^{31}P-NMR

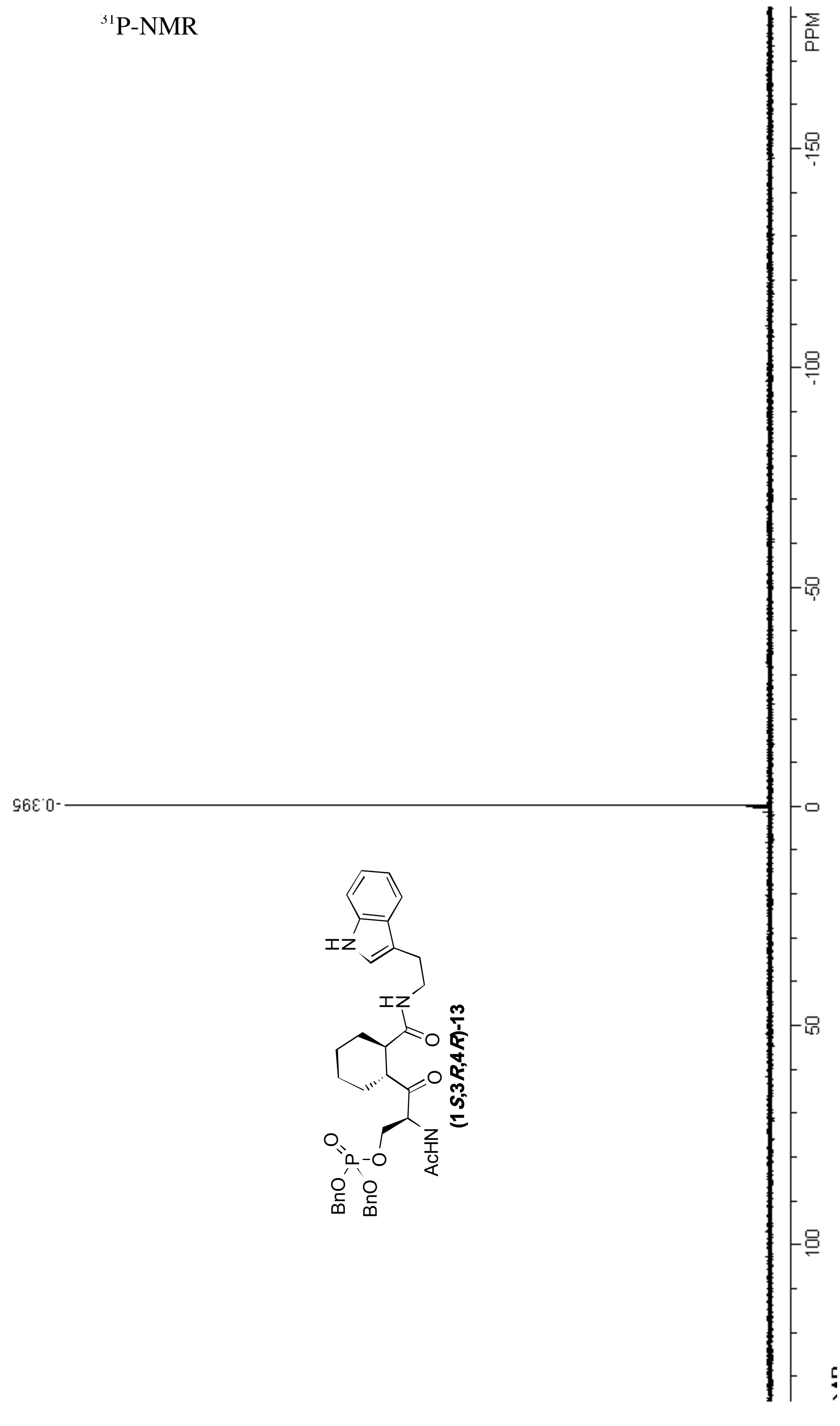

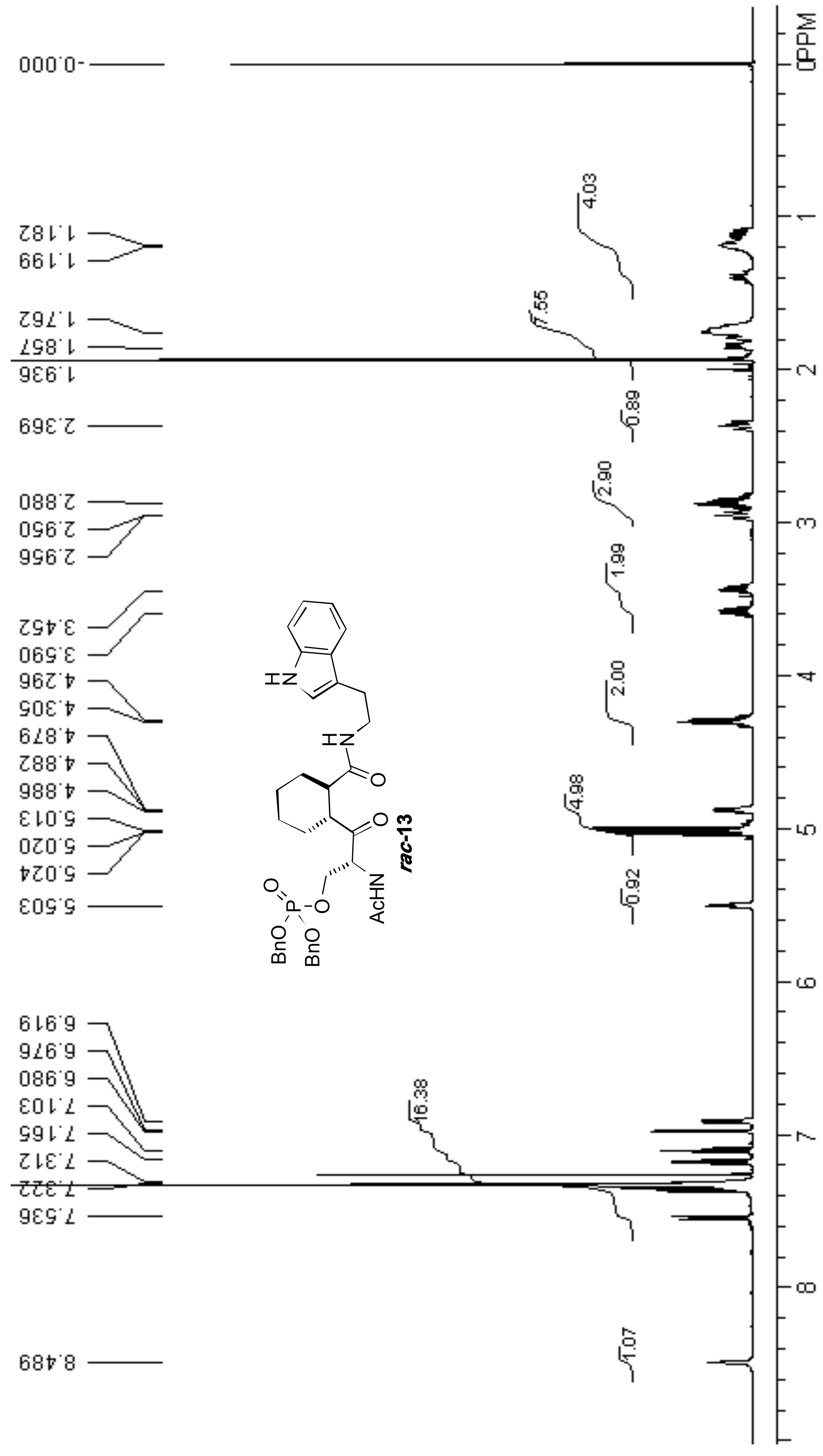

-0.000
1.182
1.199
1.762
1.857
1.936
2.369
2.880
2.950
2.956
3.452
3.590
4.296
4.305
4.879
4.882
4.886
5.013
5.020
5.024
5.503
6.919
6.976
6.980
7.103
7.165
7.312
7.322
7.536
8.489
4.03
7.55
0.89
2.90
1.99
2.00
4.98
0.92
16.38
1.07
PPM
rac-13
AcHN
BnO

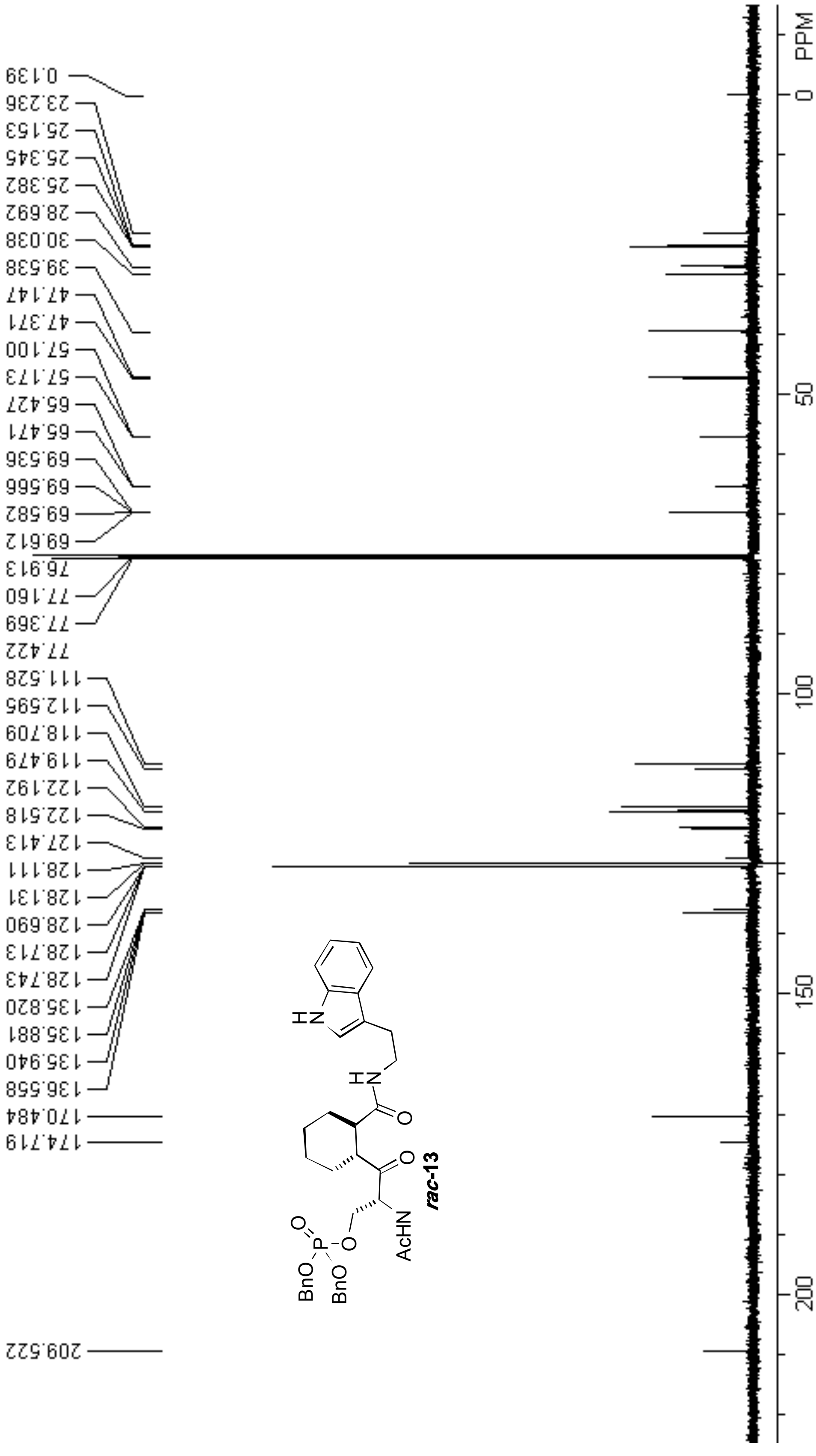
0.139
23.236
25.153
25.345
25.382
28.692
30.038
39.538
47.147
47.371
57.100
57.173
65.427
65.471
69.536
69.566
69.582
69.612
76.913
77.160
77.369
77.422
111.528
112.595
118.709
119.479
122.192
122.518
127.413
128.111
128.131
128.690
128.713
128.743
135.820
135.881
135.940
136.558
170.484
174.719
209.522
PPM
0
50
100
150
200
BnO
BnO
AcHN
rac-13

^{31}P-NMR

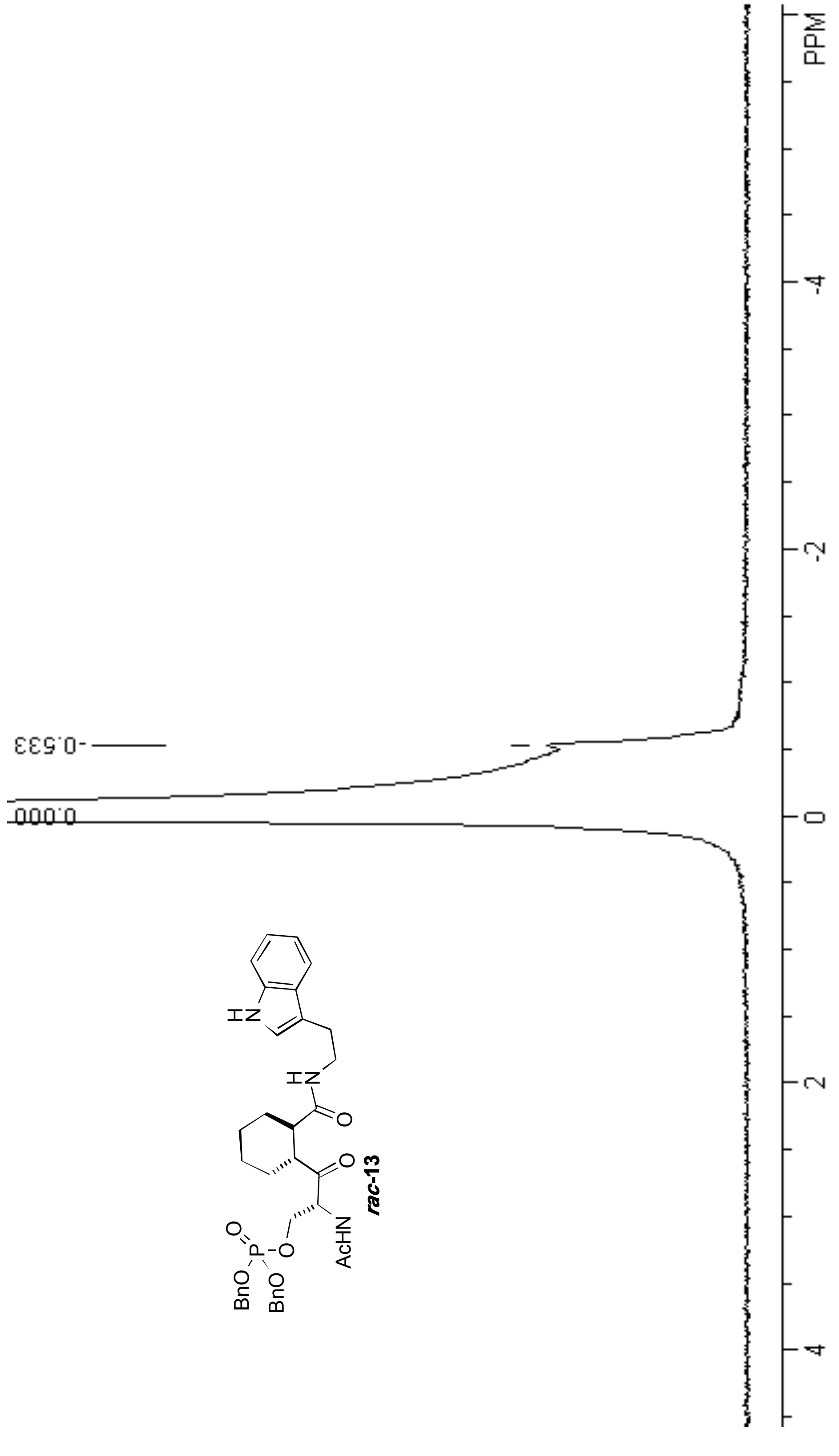

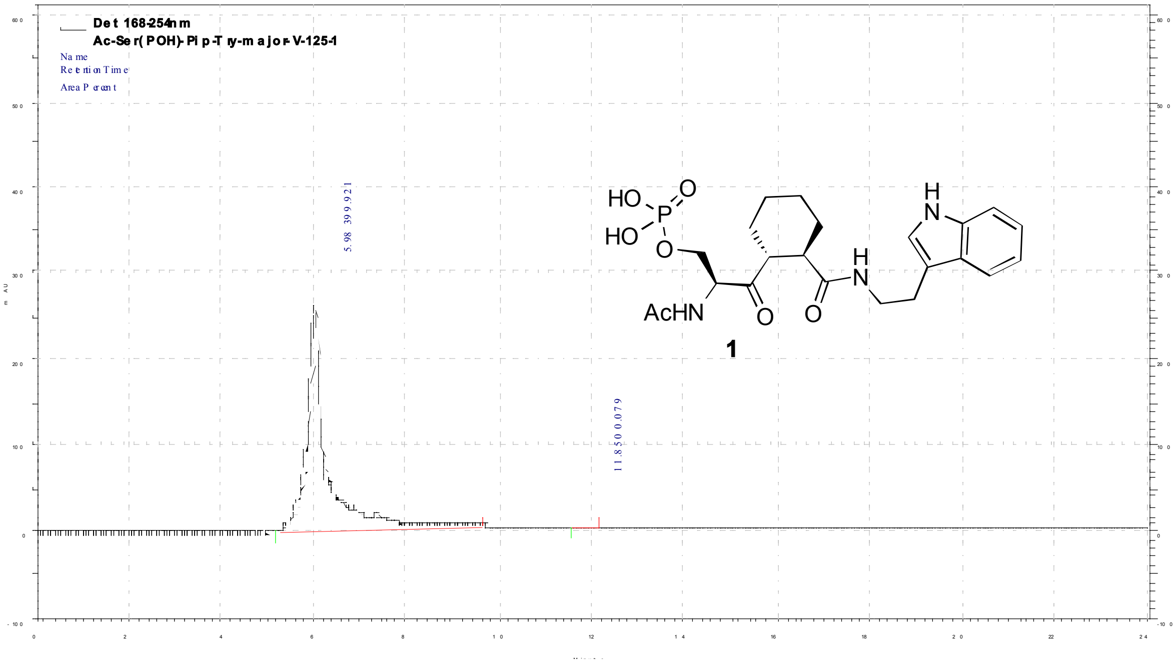
Det 168-254nm
Ac-Ser(POH)-Pip-Try-major-V-125-1
Name
Retention Time
Area Percent
5.98 399.921
11.850 0.079
HO
HO
P
O
O
O
AcHN
O
O
H
N
H
N
1
mAU

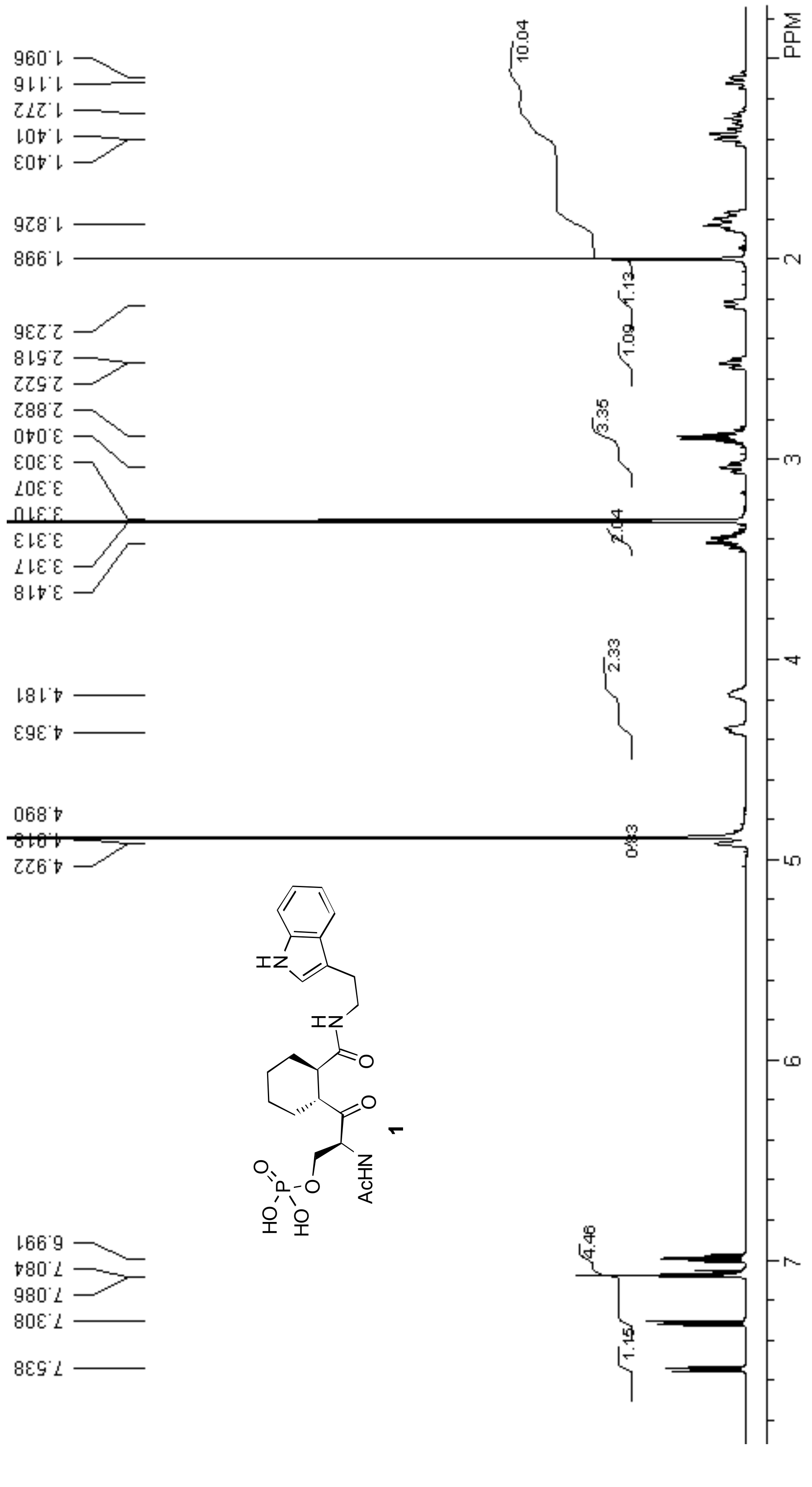
PPM
1.096
1.116
1.272
1.401
1.403
1.826
1.998
2.236
2.518
2.522
2.882
3.040
3.303
3.307
3.313
3.317
3.418
4.181
4.363
4.890
4.922
6.991
7.084
7.086
7.308
7.538
10.04
1.13
1.09
3.35
2.04
2.33
4.46
1.15
AcHN
1

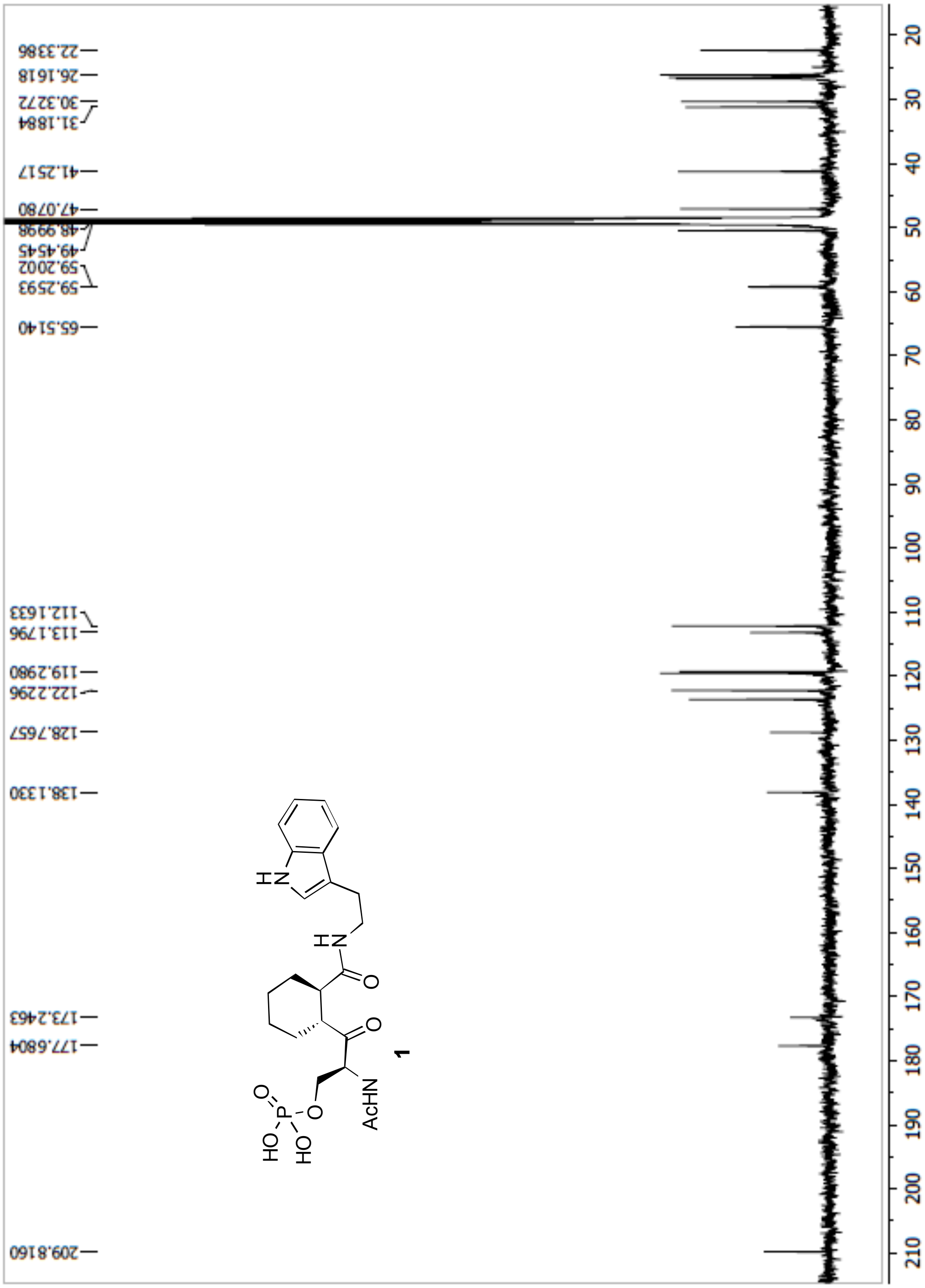
22.3386
26.1618
30.3272
31.1884
41.2517
47.0780
48.9998
49.4545
59.2002
59.2593
65.5140
112.1633
113.1796
119.2980
122.2296
128.7657
138.1330
173.2463
177.6804
209.8160
AcHN
HO
1

^{31}P-NMR

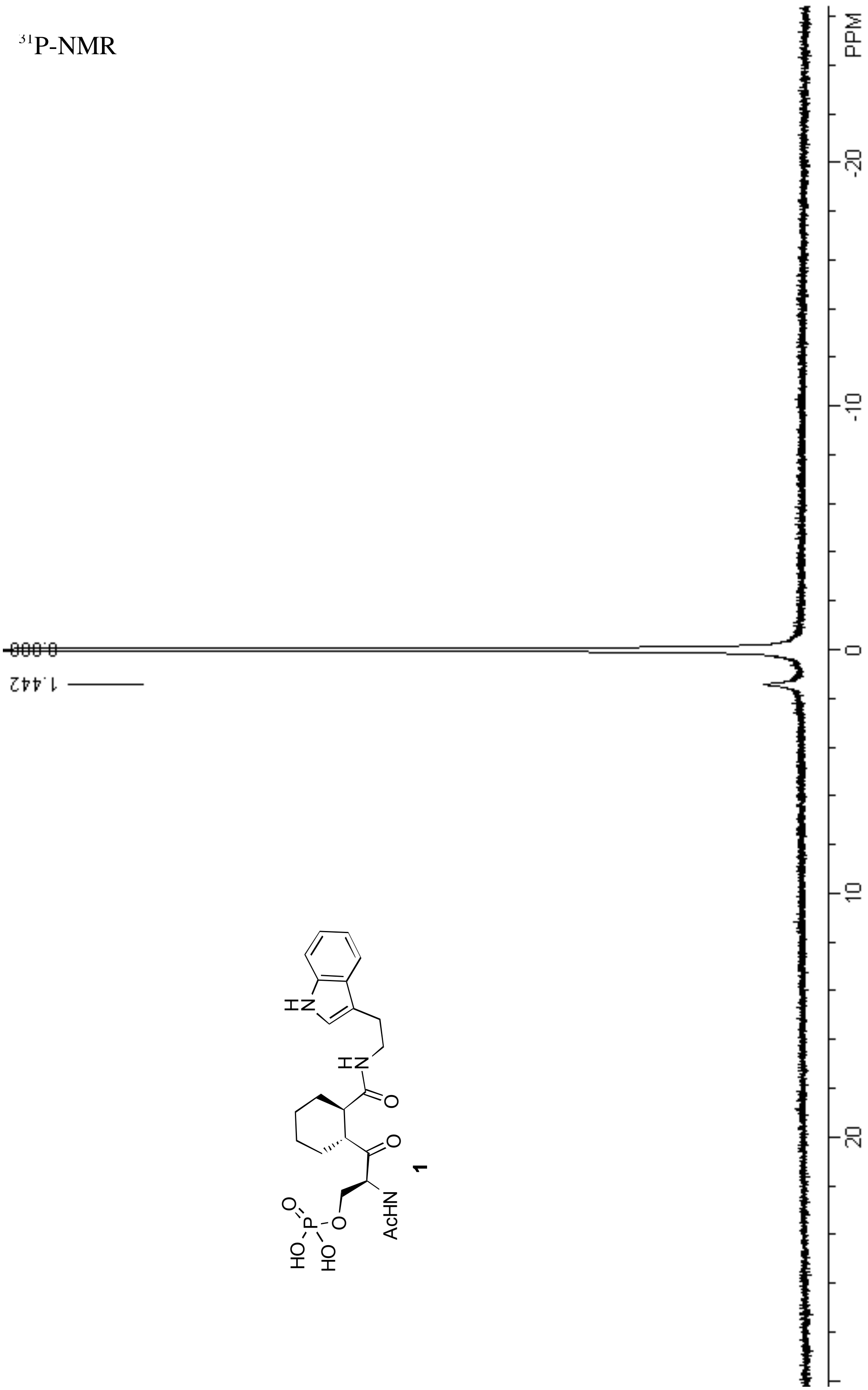

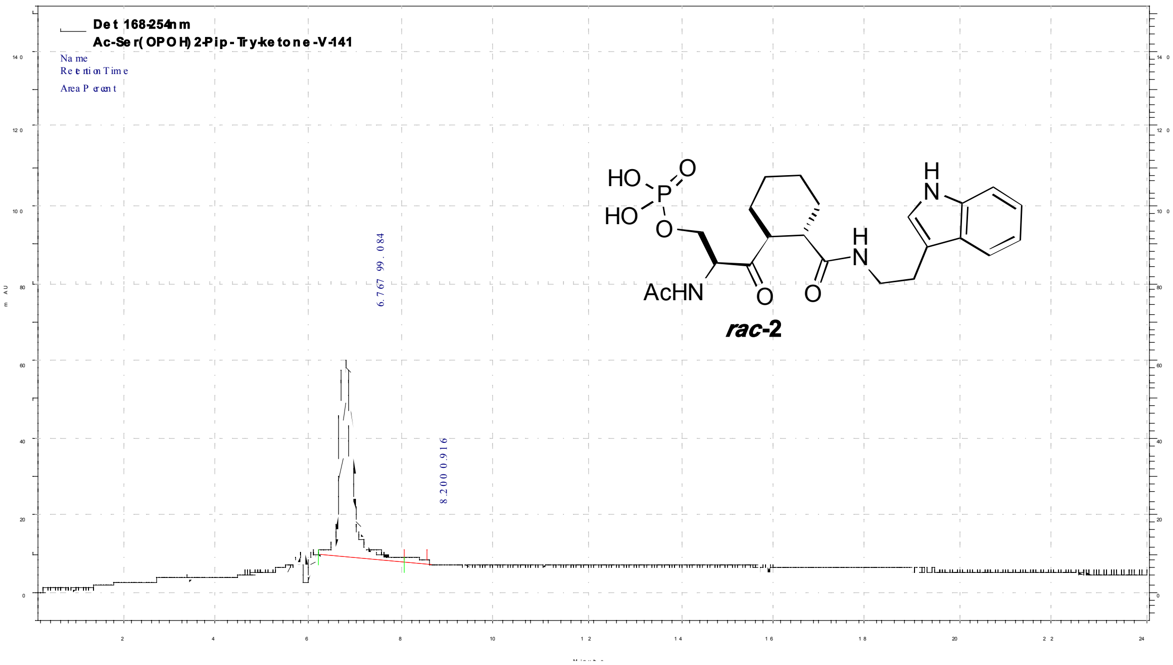
Det 168-254nm
Ac-Ser(OPOH)2-Pip-Try-ketone-V-141
Name
Retention Time
Area Percent
6.767 99.084
8.200 0.916
HO
HO
P
O
O
O
AcHN
O
O
H
N
H
N
rac-2

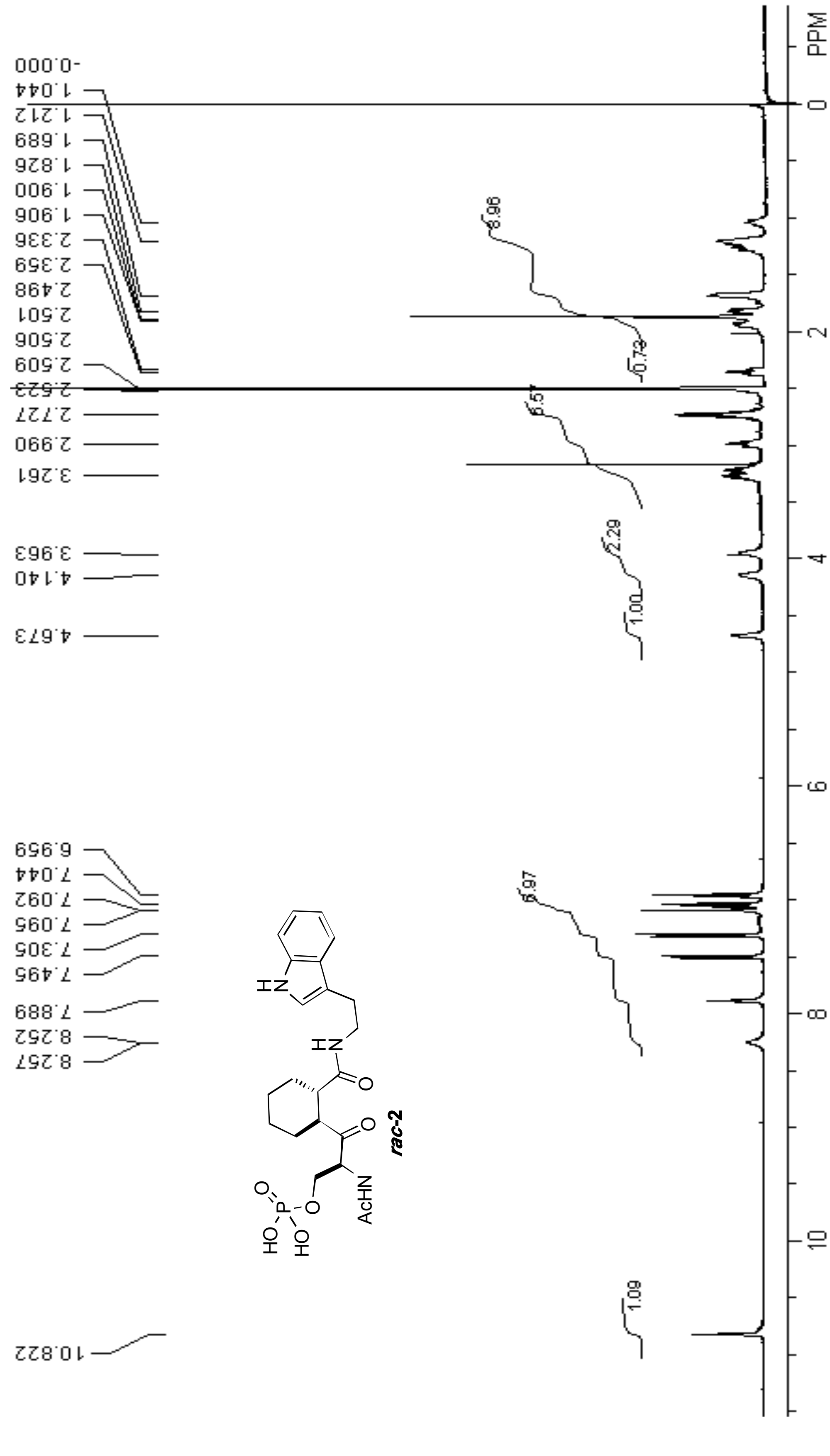
PPM
0
2
4
6
8
10
-0.000
1.044
1.212
1.689
1.826
1.900
1.906
2.336
2.359
2.498
2.501
2.506
2.509
2.523
2.727
2.990
3.261
3.963
4.140
4.673
6.959
7.044
7.092
7.095
7.305
7.495
7.889
8.252
8.257
10.822
8.96
0.73
6.57
2.29
1.00
6.97
1.09
AcHN
HO
O
P
NH
N
H
rac-2

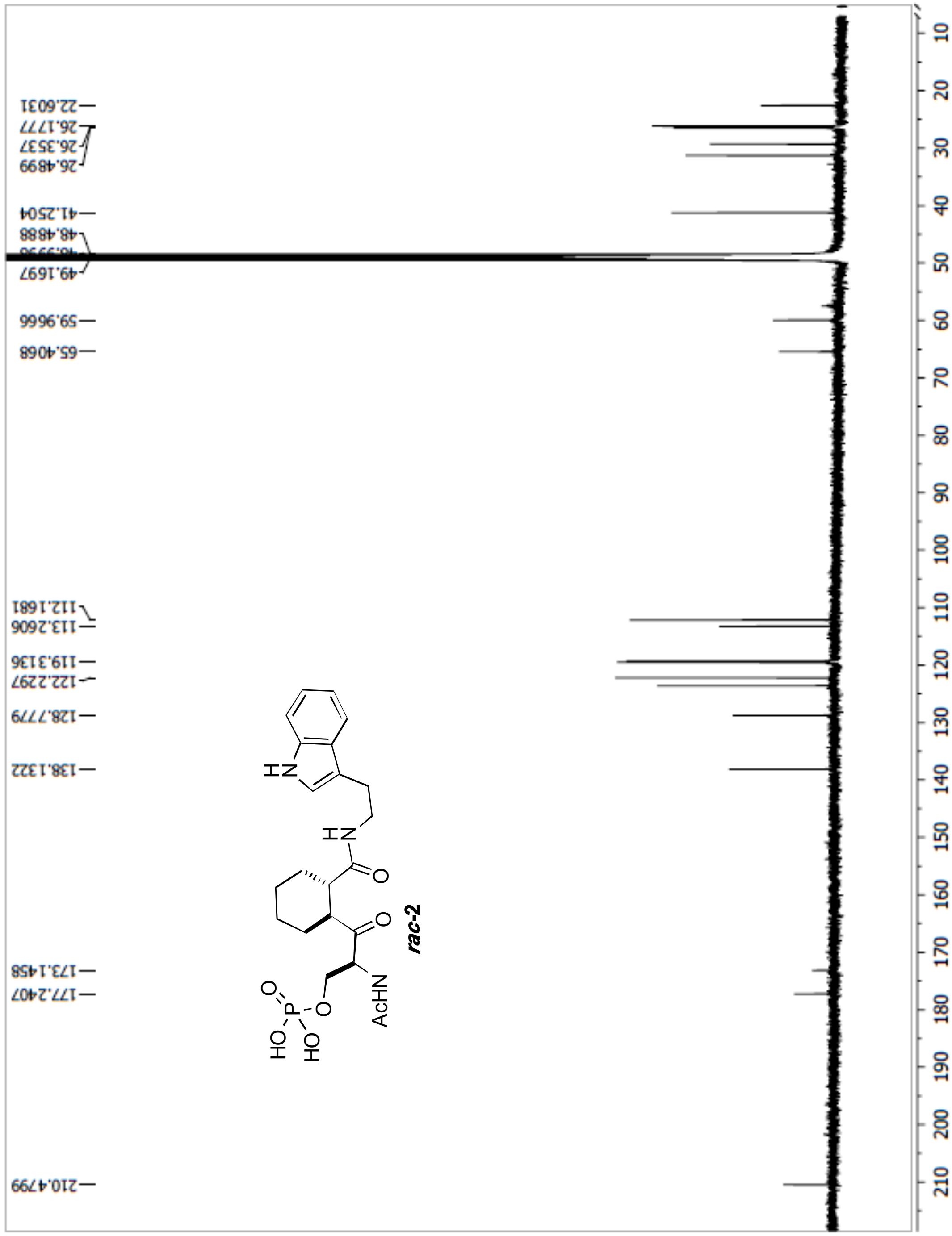
22.6031
26.1777
26.3537
26.4899
41.2504
48.4888
49.1697
59.9666
65.4068
112.1681
113.2606
119.3136
122.2297
128.7779
138.1322
173.1458
177.2407
210.4799
rac-2

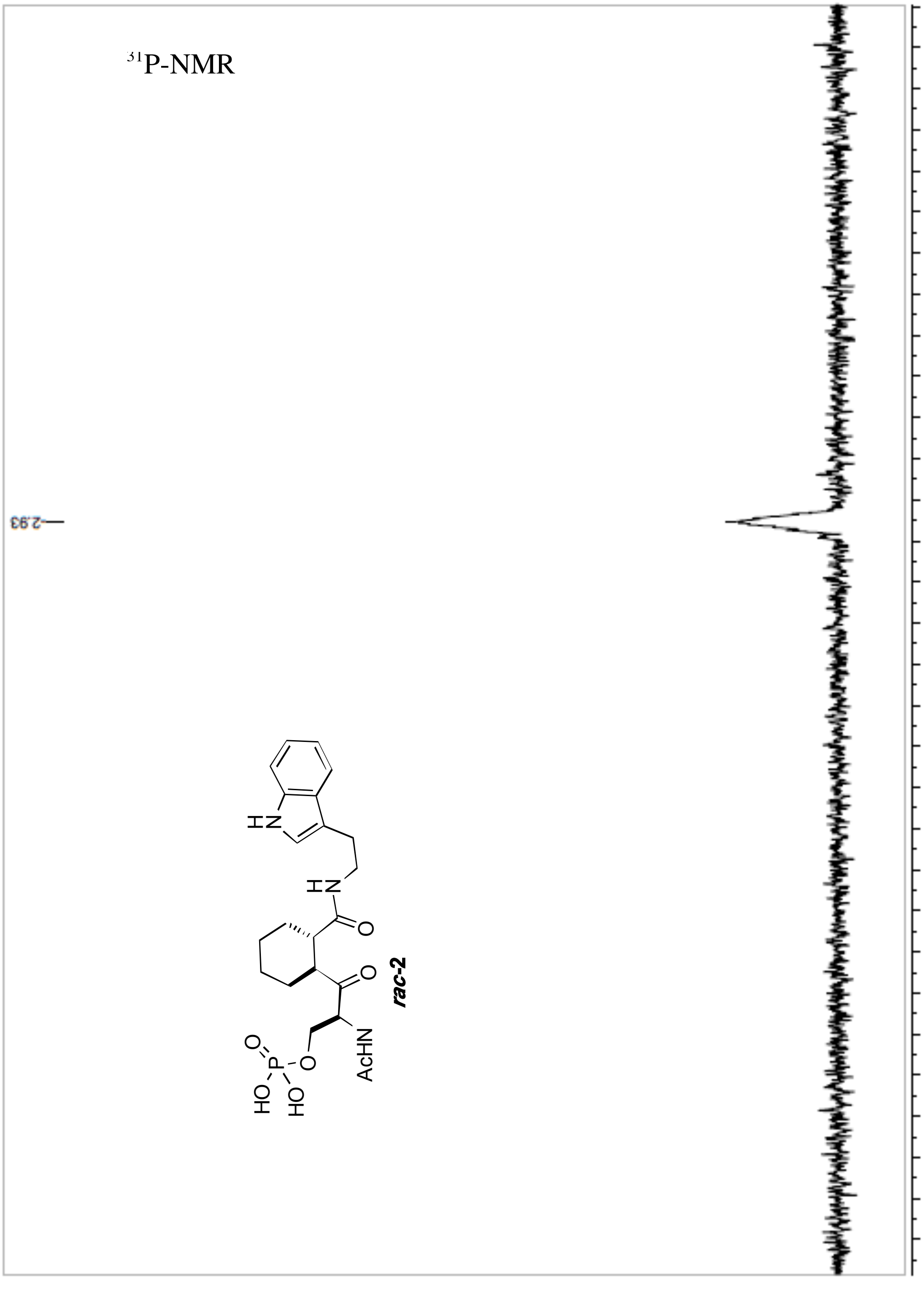
31P-NMR
-2.93
f1 (ppm)
rac-2
AcHN
HO
HO
