# Supplementary material for: Cyclohexyl Ketone Inhibitors of Pin1 Dock in a Trans-Diaxial Cyclohexane Conformation
Source: PLoS One. 2012 Sep 19;7(9):e44226. doi: 10.1371/journal.pone.0044226 (PMC3446931; doi:10.1371/journal.pone.0044226)
Supplement: Dataset S2 — Pin1 inhibition plots for 1 and rac -2. Crystallographic data, CCDC 782064 and 782063 for (1 S ,3 R ,4 R )-11 and rac -11 respectively, can be obtained free of charge from The Cambridge Crystallographic Data Centre via www.ccdc.cam.ac.uk/data_request/cif. (PDF) [file pone.0044226.s002.pdf]

Supporting Information

# Cyclohexyl ketone inhibitors of Pin1 dock in a *trans*-diaxial cyclohexane conformation

Guoyan G. Xu, Carla Slebodnick, and Felicia A. Etzkorn*

*Department of Chemistry, Virginia Tech, Blacksburg, VA 24061-0212, USA*

**Dataset S2.** Pin1 inhibition plots and curve fits for compounds **1** and ***rac*-2.**

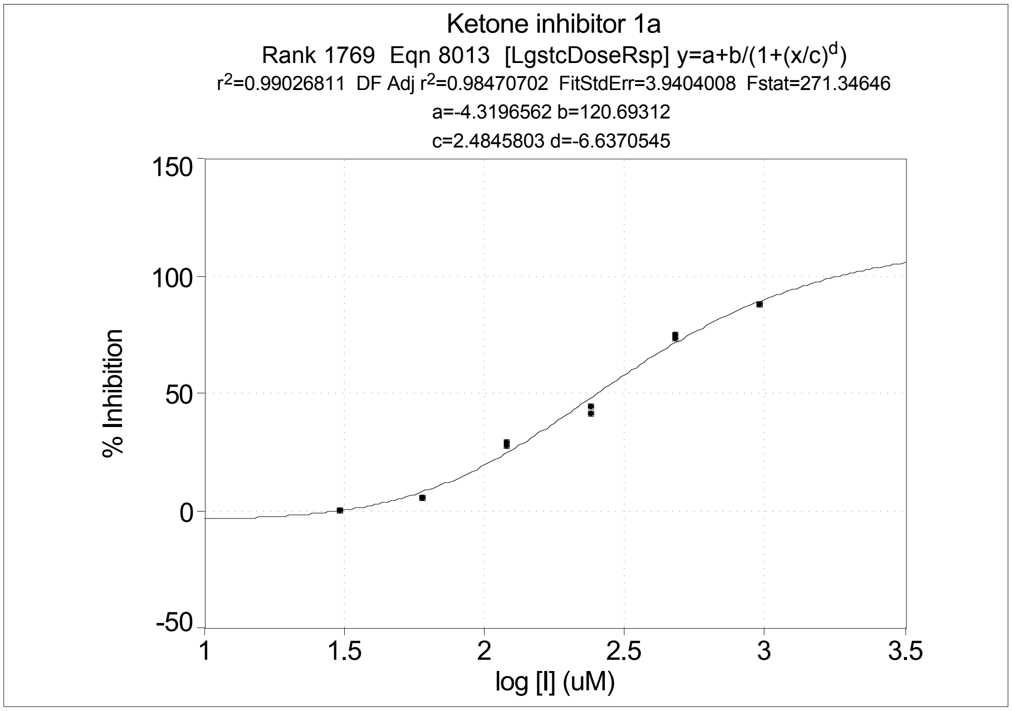


Dose response curve for inhibition of Pin1 by compound **1**. $IC_{50}$ = 260 ± 30 μM

* Corresponding author. Phone: +1 540 231 2235; fax: +1 540 231 3255; e-mail: fetzkorn@vt.edu

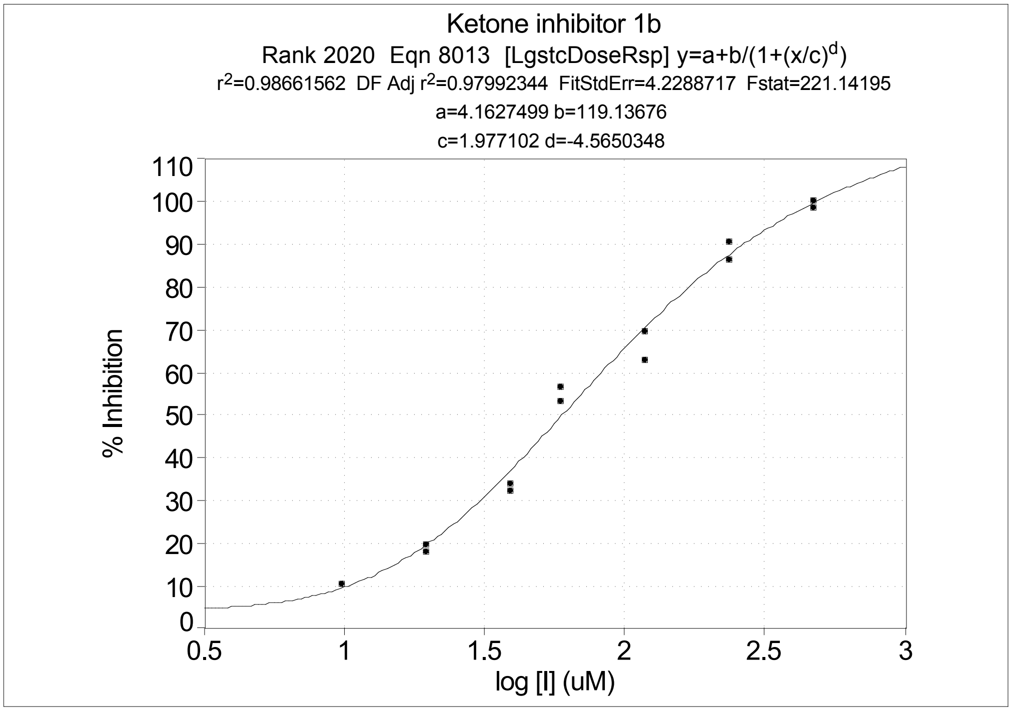


Dose response curve for inhibition of Pin1 by compound ***rac*-2**. $IC_{50}$ = 60 ± 8 μM.
